# Pediatric post-discharge mortality in resource-poor countries: a systematic review and meta-analysis

**DOI:** 10.1016/j.eclinm.2023.102380

**Published:** 2023-12-21

**Authors:** Martina Knappett, Vuong Nguyen, Maryum Chaudhry, Jessica Trawin, Jerome Kabakyenga, Elias Kumbakumba, Shevin T. Jacob, J. Mark Ansermino, Niranjan Kissoon, Nathan Kenya Mugisha, Matthew O. Wiens

**Affiliations:** a Institute for Global Health, BC Children’s Hospital and BC Women’s Hospital + Health Centre, 305-4088 Cambie Street, Vancouver, BC V5Z 2X8, Canada; b Maternal Newborn & Child Health Institute, Mbarara University of Science and Technology, Mbarara, Uganda; c Faculty of Medicine, Dept of Community Health, Mbarara University of Science and Technology, Mbarara, Uganda; d Dept of Paediatrics and Child Health, Mbarara University of Science and Technology, Mbarara, Uganda; e Walimu, Plot 5-7, Coral Crescent, Kololo, P.O. Box 9924, Kampala, Uganda; f Dept of Clinical Sciences, Liverpool School of Tropical Medicine, Liverpool, United Kingdom; g Dept of Anesthesia, Pharmacology & Therapeutics, University of British Columbia, 217-2176 Health Sciences Mall, Vancouver, BC V6T 1Z3, Canada; h BC Children’s Hospital Research Institute, 938 West 28th Ave, Vancouver, BC V5Z 4H4, Canada; i Dept of Pediatrics, BC Children’s Hospital, University of British Columbia, Rm 2D19, 4480 Oak Street, Vancouver, BC V6H 3V4, Canada

**Keywords:** Post-discharge mortality, Child mortality, Meta-analysis, Global health, Child health

## Abstract

**Background:**

Under-five mortality remains concentrated in resource-poor countries. Post-discharge mortality is becoming increasingly recognized as a significant contributor to overall child mortality. With a substantial recent expansion of research and novel data synthesis methods, this study aims to update the current evidence base by providing a more nuanced understanding of the burden and associated risk factors of pediatric post-discharge mortality after acute illness.

**Methods:**

Eligible studies published between January 1, 2017 and January 31, 2023, were retrieved using MEDLINE, Embase, and CINAHL databases. Studies published before 2017 were identified in a previous review and added to the total pool of studies. Only studies from countries with low or low-middle Socio-Demographic Index with a post-discharge observation period greater than seven days were included. Risk of bias was assessed using a modified version of the Joanna Briggs Institute critical appraisal tool for prevalence studies. Studies were grouped by patient population, and 6-month post-discharge mortality rates were quantified by random-effects meta-analysis. Secondary outcomes included post-discharge mortality relative to in-hospital mortality, pooled risk factor estimates, and pooled post-discharge Kaplan–Meier survival curves. PROSPERO study registration: #CRD42022350975.

**Findings:**

Of 1963 articles screened, 42 eligible articles were identified and combined with 22 articles identified in the previous review, resulting in 64 total articles. These articles represented 46 unique patient cohorts and included a total of 105,560 children. For children admitted with a general acute illness, the pooled risk of mortality six months post-discharge was 4.4% (95% CI: 3.5%–5.4%, I^2^ = 94.2%, n = 11 studies, 34,457 children), and the pooled in-hospital mortality rate was 5.9% (95% CI: 4.2%–7.7%, I^2^ = 98.7%, n = 12 studies, 63,307 children). Among disease subgroups, severe malnutrition (12.2%, 95% CI: 6.2%–19.7%, I^2^ = 98.2%, n = 10 studies, 7760 children) and severe anemia (6.4%, 95% CI: 4.2%–9.1%, I^2^ = 93.3%, n = 9 studies, 7806 children) demonstrated the highest 6-month post-discharge mortality estimates. Diarrhea demonstrated the shortest median time to death (3.3 weeks) and anemia the longest (8.9 weeks). Most significant risk factors for post-discharge mortality included unplanned discharges, severe malnutrition, and HIV seropositivity.

**Interpretation:**

Pediatric post-discharge mortality rates remain high in resource-poor settings, especially among children admitted with malnutrition or anemia. Global health strategies must prioritize this health issue by dedicating resources to research and policy innovation.

**Funding:**

No specific funding was received.

**Research in context**

**Evidence before this study**

Growing evidence demonstrates that children who are discharged from hospital in resource-poor settings remain highly vulnerable in the post-discharge period. Recent estimates indicate that approximately half of all deaths related to hospital admissions in these regions occur within six months after discharge. We searched MEDLINE, Embase, and CINAHL databases between 2017 and 2023 for studies conducted in countries with low or low-middle Socio-Demographic Index with a post-discharge observation period greater than seven days and supplemented these findings with studies identified in a prior systematic review published in 2018. Since the last review, the available literature on post-discharge mortality has nearly doubled, emphasizing the growing interest in this area of research. Only one meta-analysis, conducted in 2022, has compared the pooled risks of post-discharge mortality between patients with severe anemia and other health conditions in malaria-endemic Africa. This study concluded that children who were recently discharged from the hospital after recovering from severe anemia or severe acute malnutrition face an excess risk of mortality.

**Added value of this study**

This systematic review and meta-analysis aimed to quantify the burden of pediatric post-discharge mortality and associated risk factors across resource-poor countries. Data in our analysis span Africa, South-East Asia, and South America, shedding light on the global scale of this critical health issue. Secondary to a notable expansion of research over the past 5 years, pooled estimates of mortality rates across several key population subgroups are now possible, including general acute illness, pneumonia, malaria, severe malnutrition, severe anemia, diarrhea, and HIV. The evidence from this study suggests that approximately one in 20 children with general acute illness die within six months following discharge, and this proportion rises to one in 10 for children admitted with severe anemia, severe malnutrition, or HIV.

**Implications of all the available evidence**

These results highlight the significant magnitude of post-discharge mortality and underscore the urgent need for targeted interventions and improved post-discharge care. By understanding the specific risk factors associated with different subgroups, healthcare systems and policymakers can develop strategies to address these vulnerabilities and reduce post-discharge mortality rates among children.

## Introduction

More than 80% of global deaths in children occur in Sub-Saharan Africa and Southern Asia. These deaths are mostly due to preventable causes such as infectious diseases (including malaria, pneumonia, or diarrheal diseases) and are largely due to global inequities in resources and access to quality care.^1^ What is becoming apparent is that a substantial number of these deaths occur after discharge from a health facility, most occur at home or in transit seeking care, and the majority occur within six months post-discharge.^2^, ^3^, ^4^ Post-discharge deaths have been largely ignored at both policy and healthcare systems levels, and it is apparent that an urgent, concerted effort toward addressing this period of vulnerability is required in order to meet the 2030 Sustainable Development Goal neonatal and child mortality targets.^5^

The reason why efforts to address post-discharge deaths have not garnered an equivalent level of attention among policy makers and other key stakeholders, in comparison to deaths occurring in health facilities, is multifaceted. One important factor for the lack of attention is the fragmented nature of current research which has failed to consider specific population subgroups (e.g., malnutrition, anemia, general admissions) and the heterogeneous nature of the outcome (e.g., post-discharge time frame, post-discharge mortality compared to in-hospital mortality). Prior systematic reviews on this topic have been constrained by limited data, precluding the possibility of robust meta-analytic pooling within key disease and outcome subgroups.^6^^,^^7^ A significant recent expansion of research now provides a more granular understanding of pediatric post-discharge mortality, primarily as it relates to specific disease subgroups or specific risk factors. This now offers an opportunity to systematically analyze and dissect issues relating to post-discharge mortality. This insight can in turn encourage new research efforts to further guide policy and practice decisions aimed at reducing preventable deaths among children. Therefore, the objective of this systematic review and meta-analysis is to update the existing body of evidence regarding the burden and associated risk factors of pediatric post-discharge mortality following acute illness in resource-poor settings.

## Methods

This systematic review and meta-analysis was conducted in accordance with the Preferred Reporting Items for Systematic Review and Meta-analysis (PRISMA) guidelines^8^ (Appendix pp 47–50) and is registered in the International Prospective Register of Systematic Reviews (PROSPERO) (#CRD42022350975). The study protocol was peer-reviewed and published.^9^

### Search strategy and selection criteria

A systematic literature search of EMBASE, MEDLINE, and CINAHL databases was performed to identify eligible studies published between January 1, 2017 and January 31, 2023. Studies published from inception to 2017 were identified in an earlier publication,^7^ and the eligibility criteria for the present review were applied retrospectively before the addition of all remaining eligible references to the final updated pool of studies. Search strategies for each database were created in consultation with two academic librarians and were similar to the search strategies used in the previous review (Appendix pp 3–14). Briefly, subject headings related to post-discharge mortality, such as “hospitalization” and “mortality” were exploded and supplemented by keywords such as “hospital∗” or “death∗”. Subject headings and keywords pertaining to longitudinal or follow-up studies were also included. Previously published search filters for low- and middle-income countries (LMICs)^10^^,^^11^ were added and modified to include only the names of eligible countries to increase the specificity of the search. Validated pediatric search filters specific to each database were then included for capturing the population of interest.^12^ No language restrictions were made. Lastly, the reference lists of all included studies were reviewed to identify any potentially eligible studies not captured in the systematic search.

Selection criteria were set in accordance with the CoCoPop framework,^13^ a method suitable for designing a review addressing prevalence or incidence. This framework guides the eligibility criteria according to three domains of condition, context, and population. The population of interest was pediatric patients 0–18 years of age discharged from hospitals following acute illness (e.g., pneumonia, malaria, diarrheal disease). Studies reporting on patients discharged following all-cause admissions were also included under this criterion, since most admissions within these regions are secondary to acute illnesses. Studies were excluded if they did not report distinguishable pediatric data, if the patient population was discharged from a health facility not publicly accessible (e.g., military hospitals), or if they represented a specific disease population where post-discharge care and outcomes would likely differ than that of an acute illness (e.g., admissions for specific congenital diseases, cancer, surgical populations, trauma, kidney disease, cardiac disease, ophthalmic disease, sickle cell disease, liver disease, epilepsy, asthma, or prematurity). The condition was then defined as mortality following discharge from a health facility admission due to an acute illness. In terms of context, studies were included if discharges were from hospitals in resource-poor countries, defined as countries classified by the United Nations Development Programme under low or low-middle Socio-Demographic Index (SDI) quintiles in 2019^14^, ^15^, ^16^ (Appendix pp 15–18). Eligible study designs included randomized controlled trials (RCTs), cohort studies, including those conducted using surveillance or registry data, as well as case-control designs that included longitudinal arms.

Studies were excluded if they did not report post-discharge outcomes beyond 7 days, if the discharge was following a non-admission (e.g., following birth), or if the sample size was less than n = 100 patients.

### Study selection and data extraction

Two reviewers (MK and MC) independently screened titles, abstracts, and full texts against the study criteria. Any conflicts were resolved by the Principal Investigator (MW), and duplicate records were removed. In cases where several publications represented the same study cohort of participants, these publications were grouped together, and the study reporting the most comprehensive post-discharge mortality data was selected as the primary publication.

Data were extracted using a standard data extraction form developed by the review authors and the data previously extracted in the prior systematic review were reviewed for accuracy against the original papers. Seven population sub-groups were prespecified for categorization of the included studies: general acute illness, moderate and/or severe malnutrition, malaria, severe anemia, respiratory infections, and diarrheal diseases. For RCTs, if no significant difference was observed in post-discharge mortality between the control and intervention arm(s), data were extracted for both groups; if a significant difference was present, only the control group was extracted. Kaplan–Meier survival curves, where provided, were extracted using a plot digitizer.^17^

### Quality assessment

The methodological quality and risk of bias in included studies was evaluated using a modified version of the Joanna Briggs Institute (JBI) critical appraisal tool for prevalence studies.^18^ Modification of this tool involved adjusting the originally dichotomous “Yes” or “No” scale to a quantifiable scale rated as 1, 2, or 3 to assess a low, medium, or high risk of bias. Application of this tool was done twice independently by two reviewers (MK and MC), and any discrepancies were subsequently reconciled.

### Data analysis

The primary outcome of this study was the pooled prevalence of pediatric 6-month post-discharge mortality in resource-poor settings. This time point was chosen as the primary outcome since 1) it is the most common time point used in studies focusing specifically on post-discharge mortality, and 2) it represents a period of risk that is potentially amenable to novel interventional approaches. Secondary outcomes included post-discharge mortality relative to in-hospital mortality, pooled estimates of risk factors, and pooled post-discharge mortality Kaplan–Meier survival curves.

Fixed and random effects meta-analyses were conducted to estimate overall post-discharge mortality at six months, and forest plots were used to illustrate 6-month mortality rates. Risk factors for post-discharge mortality, reported as hazard ratios (HRs), relative risks (RRs), or odds ratios (ORs), were pooled and analyzed separately, though only sufficient data were provided for the analysis of pooled HRs. When a study provided both adjusted and unadjusted estimates, we preferentially extracted the adjusted estimates only. Estimates from both unadjusted and adjusted analyses were pooled and analyzed using a mixed effects meta-regression model. The variance of the hazards ratios for risk factors was estimated from the 95% confidence intervals (CIs).^19^ Studies may have differed in how they addressed readmissions, with some subsequently counting an individual at risk multiple times when estimating their hazards ratios. Hazards ratios were pooled as they were analyzed and presented in the original paper.

There were many instances in which studies reported survival curves. The Combescure distribution-free approach assuming random effects was used for pooling these survival curves and to estimate the pooled risk of post-discharge mortality over time to 12 months.^20^ The number at risk at each time point for pooling survival curves was estimated according to the methods described by Tierney and colleagues^18^ using code made publicly available by Guyot and colleagues^21^ with the minimum amount of information needed being the initial sample size.

Studies had varying post-discharge follow-up ranging from 4 weeks to 12 months. For the 6-month meta-analysis for post-discharge mortality, we only included studies whose endpoint was ≥4 and <8 months and for the 12-month analysis, we only included studies whose endpoint was ≥8 months. Additionally, in several cases, authors reported survival curves for specific groups (e.g., age and disease subgroups) or had a 12-month endpoint and did not report their 6-month post-discharge mortality rate. Since all results of interest are in reference to mortality, pooled survival curves were summarized and presented as percentage mortality curves (i.e., 1 − survival proportion) rather than survival curves.

We used the mortality curves to estimate the 6-month post-discharge mortality for these groups and included them in the fixed and random-effects meta-analyses for post-discharge mortality. Where applicable, we also combined multiple curves from a single study to estimate the mortality rate for the overall cohort.

Heterogeneity was measured using the I^2^ statistic, with an I^2^ value of 25% categorized as low heterogeneity, 50% as moderate heterogeneity, and 75% as indicative of substantial heterogeneity.

We conducted a complete analysis using all studies regardless of their bias assessment. Publication bias was assessed by visual inspection of asymmetry in the funnel plots and Egger’s test for asymmetry (Appendix pp 30).

### Assumptions

If there was more than 50% of a population overlap between studies drawing from the same data and non-overlapping data were not separately reported, data were only extracted for the larger cohort. This approach assumes that the larger cohort includes the children analyzed in the smaller cohort. The denominator used for assessing post-discharge mortality included all children enrolled for follow-up at discharge, including any children lost to follow-up prior to 6 months post-discharge. The children lost to follow-up in this case were, therefore, assumed to be alive, which may underestimate post-discharge mortality rates. Studies which enrolled all acute illness admissions were used to estimate overall post-discharge mortality prevalence. For study cohorts representing individuals from a particular disease subgroup (e.g., severe anemia) only, they were included in the meta-analysis of that disease subgroup but not used in the overall general admissions post-discharge mortality estimates.

### Role of the funding source

There was no funding source for this study.

## Results

**Fig. 1:**
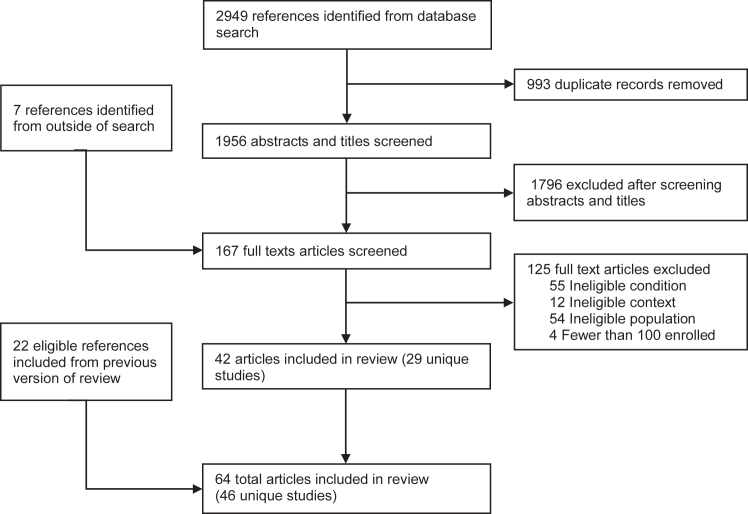
**PRISMA flowdiagram.**

Of 1963 articles identified through the systematic search, a total of 42 articles were identified. These were combined with 22 previously identified articles, resulting in a total of 64 articles eligible for inclusion in this review.^2^, ^3^, ^4^^,^^22^, ^23^, ^24^, ^25^, ^26^, ^27^, ^28^, ^29^, ^30^, ^31^, ^32^, ^33^, ^34^^,^^35^, ^36^, ^37^, ^38^, ^39^, ^40^, ^41^, ^42^, ^43^, ^44^, ^45^, ^46^, ^47^, ^48^, ^49^^,^^50^, ^51^, ^52^, ^53^, ^54^, ^55^, ^56^, ^57^, ^58^, ^59^, ^60^, ^61^, ^62^, ^63^, ^64^^,^^65^, ^66^, ^67^, ^68^, ^69^, ^70^, ^71^, ^72^, ^73^, ^74^^,^^75^, ^76^, ^77^, ^78^, ^79^, ^80^, ^81^, ^82^, ^83^ Of these 64 publications, data from 46 unique patient cohorts were represented. The study reporting the most comprehensive post-discharge mortality data was chosen as the primary publication (Fig. 1, Table 1, Appendix pp 19–23). With regards to the primary objective of measuring post-discharge mortality rates, the included studies were considered to be of reasonable quality, with the primary areas of concern being low (<90%) follow-up rates and inconsistent measurement methods for the condition or risk factors (Appendix pp 28–29). Among the seven pre-specified population sub-groups, we included studies examining acute illness (n = 15), moderate/severe malnutrition (n = 10), malaria (n = 10), severe anemia (n = 9), respiratory infections (n = 9), diarrheal diseases (n = 7). Though not pre-specified, HIV emerged as an eighth population subgroup since it was reported separately in four studies of general acute illness. The remaining two study populations remained uncategorized, with one focused on pediatric admissions secondary to SARS-CoV-2 and the other focused on admissions related to infections of the central nervous system. Most (n = 45, 97.8%) studies were conducted in Africa (n = 37), South-East Asia (n = 7), or both (n = 1). Study designs included prospective cohorts (n = 23, 50.0%), RCTs (n = 11, 23.9%), retrospective cohorts (n = 8, 17.4%), and case-control studies with longitudinal arms (n = 4, 8.7%). The follow-up period ranged from seven days to five years, though only outcomes to one year were extracted.

**Table 1:** Study characteristics.

	Study dates	Country	Study population enrolled	Population subgroups represented	Children enrolled, N	Age range	Duration of follow-up
Ashraf et al., 2012^22^	2006–2008	Bangladesh	Severe pneumonia	Respiratory infections	180	2–59 months	3 months
Berkley et al., 2016^24^^,^^40^^,^^58^^,^^83^	2009–2013	Kenya	Severe acute malnutrition	Severe malnutrition	1781	60 days–59 months	12 months
Biai et al., 2007^25^	2004–2006	Guinea-Bissau	Malaria	Malaria	951	3–60 months	28 days
Brim et al., 2017^27^^,^^49^	2012–2014	Malawi	Cerebral malaria	Malaria	258	>12 months	12 months
Bwakura-Dangarembizi et al., 2021^29^^,^^30^	2016–2018	Zambia, Zimbabwe	Severe acute malnutrition	Severe malnutrition	755	0–60 months	52 weeks
CHAIN Network, 2022^3^^,^^74^	2016–2019	Bangladesh, Burkina Faso, Kenya, Malawi, Pakistan, Uganda	Acute illness	General acute illness	3101	2–23 months	180 days
Chapagain et al., 2022^32^	2021	Nepal	COVID-19	Other	156	0–14 years	90 days
Chhibber et al., 2015^33^	2008–2012	Gambia	Pneumonia, sepsis, or meningitis	General acute illness	3952	2–59 months	180 days
Chisti et al., 2014^34^	2011–2012	Bangladesh	Severe malnutrition and radiological pneumonia	Severe malnutrition, respiratory infections	405	0–59 months	12 weeks
Grenov et al., 2017^41^	2012–2013	Uganda	Severe acute malnutrition	Severe malnutrition	400	6–59 months	8–12 weeks
Hamaluba et al., 2021^42^	2018–2019	Kenya	Uncomplicated, non-severe malaria	Malaria	217	2–12 years	42 days
Hau et al., 2018^31^^,^^43^	2014	Tanzania	All admissions	General acute illness	506	2–12 years	12 months
Hawkes et al., 2015^36^^,^^38^^,^^44^	2011	Uganda	Severe malaria	Malaria	180	1–10 years	6 months
Hennart et al., 1987^45^	1970	Zaire (Democratic Republic of the Congo)	Severe protein-energy malnutrition	Severe malnutrition	171	0–6+ years	5 years
Islam et al., 1996^46^	1991–1992	Bangladesh	Diarrhea	Diarrhea	500	1–23 months	12 weeks
Kerac et al., 2014^26^^,^^47^	2006–2007	Malawi	Malnutrition	Severe malnutrition	1024	5–168 months	1 year
Kwambai et al., 2020^48^	2016–2018	Kenya, Uganda	Severe malaria	Malaria	525	0–5 years	26 weeks
Madrid et al., 2019^2^	2000–2016	Mozambique	All admissions	General acute illness	18,023	0–15 years	90 days
Maitland et al., 2019^50^	2014–2017	Malawi, Uganda	Severe anemia	Severe anemia	3983	2 months–12 years	90 days
Masoza et al., 2022^51^	2014–2015	Tanzania	All admissions	General acute illness	525	1 month–12 years	3 months
Moisi et al., 2011^52^	2004–2008	Kenya	All admissions	General acute illness	10,277	0–15 years	1 year
Mukasa et al., 2021^53^	2003–2007	Tanzania	All admissions	General acute illness	861	<5 years	6 months
Mwangome et al., 2017^54^	2007–2014	Kenya	All admissions	General acute illness	2882	1–6 months	1 year
Mwene-Batu et al., 2020^55^	1988–2007	Democratic Republic of Congo	Severe acute malnutrition	Severe malnutrition	1981	0–144 months	Unspecified
Namazzi et al., 2022^56^	2014–2017	Uganda	Severe malaria	Malaria	598	6 months–4 years	12 months
Ngari et al., 2017^57^	2007–2012	Kenya	Admitted with or without severe pneumonia	Respiratory infections, general acute illness, HIV	7731	1–59 months	12 months
Ngari et al., 2020^80^	2007–2016	Kenya	All admissions	General acute illness, HIV	3196	60–155 months	12 months
Nkosi-Gondwe et al., 2021^61^	2016–2018	Malawi	Severe anemia	Severe anemia	375	0–5 years	15 weeks
Olupot-Olupot et al., 2014^61^	2011–2012	Uganda	Severe anemia	Severe anemia	160	60 days–12 years	28 days
Opoka et al., 2020^63^	2016–2018	Uganda	Severe anemia	Severe anemia	282	0–5 years	6 months
Ouma et al., 2020^23^^,^^37^^,^^62^^,^^64^^,^^70^	2008–2013	Uganda	Cerebral malaria and severe malarial anemia	Malaria, severe anemia	502	18 months–5 years	24 months
Page et al., 2017^65^	2009–2012	Uganda	Suspected infections of the central nervous system	Other	459	2 months–12 years	6 months
Pavlinac et al., 2021^66^^,^^67^	2016–2019	Kenya	All admissions	General acute illness	1400	1–59 months	6 months
Phiri et al., 2008^59^^,^^68^	2002–2004	Malawi	Severe anemia	Severe anemia	758	0–5 years	18 months
Phiri et al., 2012^69^	2006–2009	Malawi	Severe malarial anemia	Malaria, severe anemia	1414	4–59 months	6 months
Roy et al., 1983^71^	1979–1980	Bangladesh	Diarrhea	Diarrhea	551	3–36 months	12 months
Shahrin et al., 2020^72^	2015–2017	Bangladesh	Admitted for diarrhea and had both severe pneumonia and severe acute malnutrition	Diarrhea, respiratory infections, severe malnutrition	191	0–59 months	30 days
Stanton et al., 1986^81^	1983	Bangladesh	Diarrhea	Diarrhea	112	24–72 months	4–5 months
Talbert et al., 2019^73^	2007–2015	Kenya	Admitted with or without diarrhea	General acute illness, diarrhea, pneumonia	17,442	2–59 months	1 year
Tomczyk et al., 2019^75^	2007–2013	Guatemala	Acute respiratory illness	Respiratory infections	4109	0–2 years	6 weeks
Veirum et al., 2007^76^	1991–1996	Guinea-Bissau	All admissions	General acute illness	3373	0–6 years	365 days
Villamor et al., 2005^77^	1993–1997	Tanzania	Pneumonia	Respiratory infections	687	6–60 months	2 years
West et al., 1999^78^	1991–1994	Gambia	Acute lower respiratory tract infections	Respiratory infections	190	0–5 years	2–5 years
Wiens et al., 2015^39^^,^^79^	2012–2013	Uganda	Proven or suspected infection	General acute illness, diarrhea, malaria, severe malnutrition, respiratory infections, HIV	1307	6 months–5 years	6 months
Wiens et al., 2023^4^	2017–2020	Uganda	Suspected sepsis	General acute illness, severe anemia, diarrhea, malaria, severe malnutrition, respiratory infections, HIV	6545	0–60 months	6 months
Zucker et al., 1996^82^	1991	Kenya	Anemia	Severe anemia	584	0–5 years	8 weeks

**Fig. 2:**
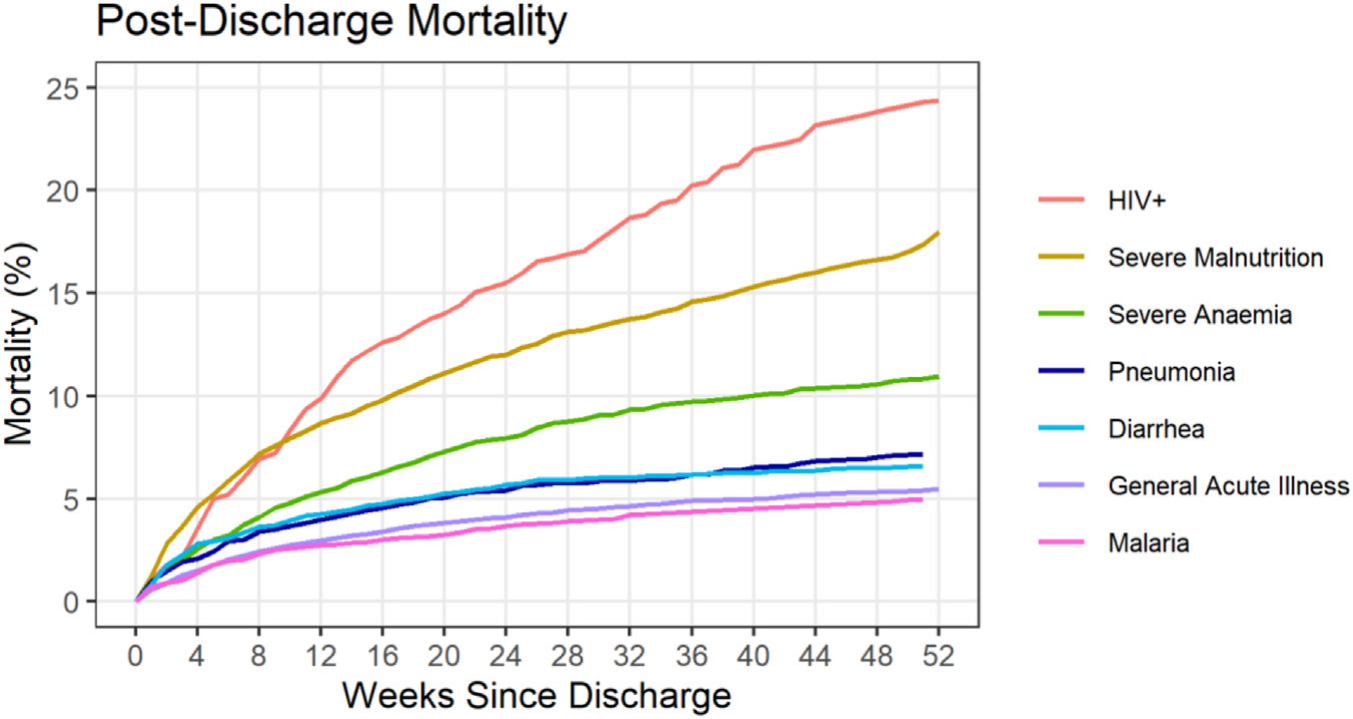
**Pooled post-discharge mortality curves (1 minus survival) by population sub-group**.

Seventeen studies (37.0%) provided survival curves for digitization and pooling, allowing for a visualization of incremental survival probability over 12 months. These curves revealed that children admitted due to malaria, diarrheal illness, acute illness, and pneumonia experienced a majority of the deaths within the first 3 months after discharge. However, children admitted with severe anemia, severe malnutrition and HIV infection had persistent mortality rates over the entire 12-month post-discharge period (Fig. 2, Appendix pp 39–40). The median time to death among these disease subgroups ranged from 3.3 to 8.5 weeks, with diarrhea having the shortest median time to death and anemia having the longest median time to death (Appendix pp 38).

**Fig. 3:**
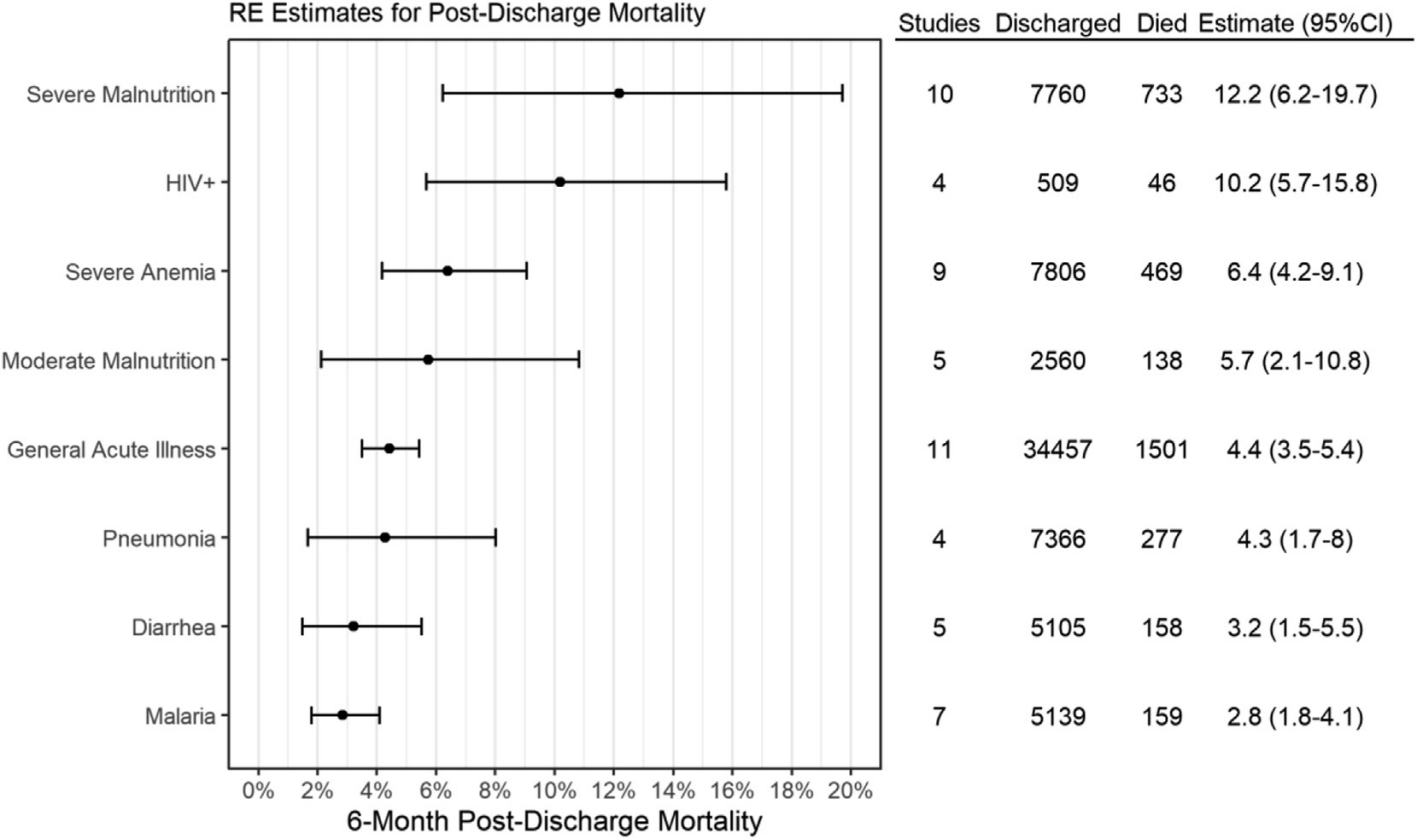
**6-month post-discharge mortality random effects estimates among population sub-groups.** Abbreviations: RE = random effects.

Among studies of children admitted with a general acute illness, the pooled risk of mortality was 2.1% (95% CI: 0.9%–3.9%, I^2^ = 96.9%, n = 3 studies, 22,371 children) at one month, 3.3% (95% CI: 2.1%–4.7%, I^2^ = 96.8%, n = 7 studies, 31,096 children) at three months, and 4.4% (95% CI: 3.5%–5.4%, I^2^ = 94.2%, n = 11 studies, 34,457 children) at six months after discharge (Fig. 3, Appendix pp 31). Six studies (n = 28,552 children) further provided follow-up data to 12 months, with a pooled post-discharge mortality risk of 5.1% (95% CI: 3.0%–7.6%) (Appendix pp 33). Using the pooled mortality curves, the estimated post-discharge mortality (95% CI) at one, three, six, and 12 months was 1.5% (0.8%–2.2%), 3.0% (1.9%–4.0%), 4.3% (2.8%–5.7%), and 5.5% (3.8%–7.1%), respectively. In comparison, the pooled in-hospital mortality rate for general acute illness admissions was slightly higher at 5.9% (95% CI: 4.2%–7.7%, n = 12 studies, 63,307 children) (Appendix pp 41–42).

Of the studies in individual disease sub-groups, the highest pooled 6-month post-discharge mortality estimates were reported in studies of children admitted with severe malnutrition (12.2%, 95% CI: 6.2%–19.7%, I^2^ = 98.2%, n = 10 studies, 7760 children), children infected with HIV (10.2%, 95% CI: 5.7%–15.8%, I^2^ = 66.5%, n = 4 studies, 509 children), and those with severe anemia (6.4%, 95% CI: 4.2%–9.1%, I^2^ = 93.3%, n = 9 studies, 7806 children) (Fig. 3, Appendix pp 32). These conditions were followed by moderate malnutrition (5.7%, 95% CI: 2.1%–10.8%, I^2^ = 94.9%, n = 5, 2560 children), pneumonia (4.3%, 95% CI: 1.7%–8.0%, I^2^ = 98.0%, n = 4 studies, 7366 children), diarrhea (3.2%, 95% CI: 1.5%–5.5%, I^2^ = 90.3%, n = 5 studies, 5139 children), and malaria (2.8%, 95% CI: 1.8%–4.1%, I^2^ = 81.7%, n = 7 studies, 5139 children). This trend remained relatively consistent among the 12-month post-discharge pooled estimates, with the estimated post-discharge mortality rate of HIV-infected children reaching 18.0% (95% CI: 13.3%–23.0%, I^2^ = 94.5%, n = 2 studies, 364 children) at 12 months post-discharge (Appendix pp 33–34). Small changes were observed in the relative ranking of disease subgroups between the pooled mortality curves and the pooled risk estimates, such as between HIV infection and severe malnutrition. To determine if the presence of RCTs or data predating 2010 had a substantial impact on the summary estimates of our primary analysis, we conducted two sensitivity analyses excluding these studies. Neither analysis found any substantial change in our 6-month mortality estimates (Appendix pp 46).

**Fig. 4:**
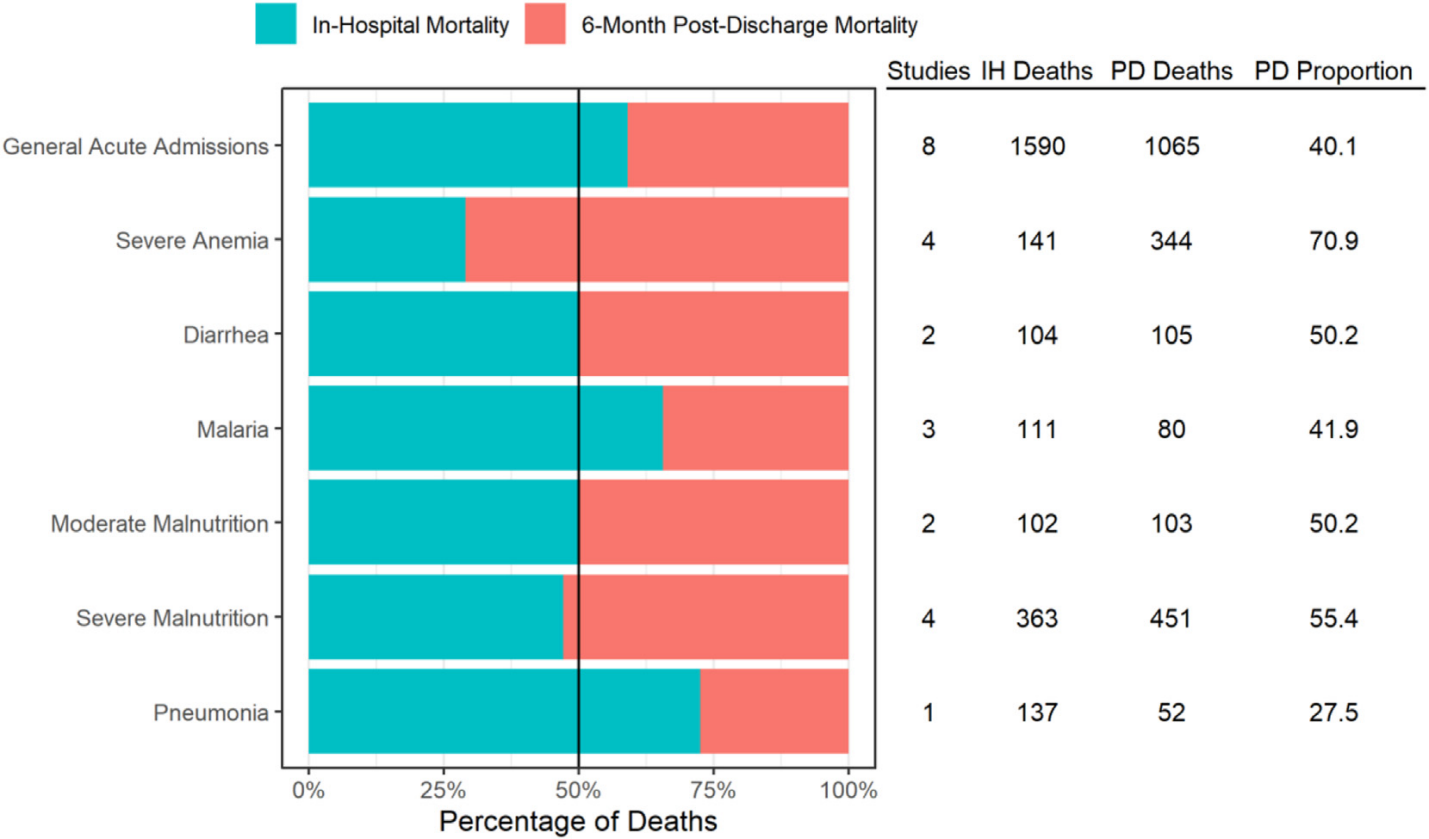
**Percentage of total deaths among population sub-groups, in-hospital versus six-month post-discharge.** Abbreviations: IH = in-hospital; PD = post-discharge.

Among studies which measured deaths occurring both during and after the hospitalization period, post-discharge deaths accounted for a significant proportion of overall deaths, but this was particularly high among children admitted with either severe anemia or severe malnutrition, where the post discharge period contributed to 70.9% (95% CI: 66.9%–75.0%) and 55.4% (95% CI: 52.0–58.8%) of total deaths, respectively (Fig. 4, Appendix pp 43–45). The post-discharge mortality proportion relative to in-hospital mortality was lowest for pneumonia (27.5%), though this was only based on results from one study.

**Fig. 5:**
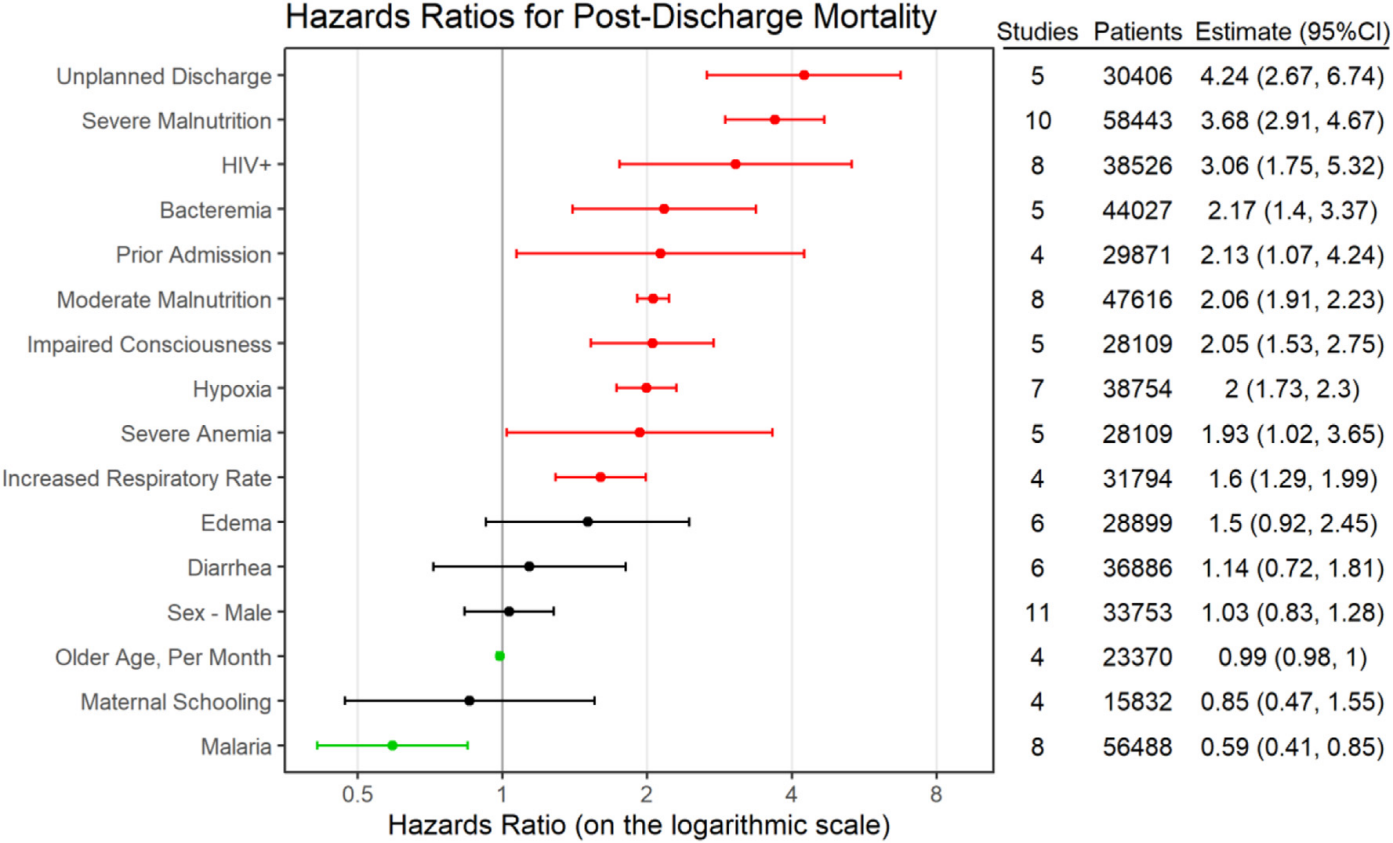
**Pooled hazard ratios (random-effects estimate) for post-discharge mortality across all population sub-groups.** Red: indicates confidence interval did not include 1 and increased the risk of post-discharge mortality. Green: indicates confidence interval did not include 1 and reduced risk of mortality. Black: indicates confidence interval includes 1 and the effect on mortality is inconclusive. Note: Increased respiratory rate included studies that defined it as a respiratory rate >30 breaths per minute or tachypnea. The number of patients was estimated based on the number of children discharged.

Overall, 23 studies measured and reported risk factors for post-discharge mortality (Fig. 5; Appendix 35–37). Unplanned discharges (i.e., absconded) (HR: 4.24, 95% CI: 2.67, 6.74, n = 5 studies, 30,406 children), severe malnutrition (HR: 3.68, 95% CI: 2.91–4.67, n = 10 studies, 58,443 children), and HIV seropositivity (HR: 3.06, 95% CI: 1.75–5.32, n = 8 studies, 38,526 children) contributed the greatest risk. These were followed by bacteremia, prior admission, moderate malnutrition, impaired consciousness, hypoxia, severe anemia and increased respiratory rate. Conversely, protective factors were older age (HR: 0.99 per month increase in age, 95% CI: 0.98–1.00, n = 4 studies, 23,370 children) and malaria (HR: 0.59, 95% CI: 0.41–0.85, n = 8 studies, 56,488 children).

## Discussion

This systematic review and meta-analysis investigated 46 unique studies, conducted in resource-poor countries, examining pediatric post-discharge mortality. Our findings underscore the significant and unresolved global challenge posed by post-discharge mortality among children across Africa and South-East Asia, and they further demonstrate its importance within multiple disease domains. The results of this review align with previous systematic reviews and emphasize that rates of mortality that follow discharge are similar to rates observed during the initial hospitalization period.^6^^,^^7^ Despite a near doubling of data on this matter since the last published review, efforts to address this public health concern have remained inadequate. Given the substantial number of deaths occurring after discharge, the development and evaluation of interventions will be critical to addressing this largely neglected contributor to childhood mortality. Such interventions, alongside the development of approaches that target the at-risk child, will be instrumental in achieving the Sustainable Development Goal of ending preventable child deaths by 2030.

The findings from this study reaffirm that approximately one in twenty children admitted with general acute illness die within 6 months of discharge. This risk increases substantially among children with severe anemia, severe malnutrition, and HIV where as many as one in ten dies during the post-discharge period. The pooling of survival curves highlighted important differences between various disease subgroups. Most notably, visualization underscores the persistence of risk over time among malnourished and anemic children. Such observations are essential in the development of comprehensive strategies to improve long-term outcomes. Within all disease groups, admission factors reflecting illness acuity, such as hypoxia, impaired consciousness and increased respiratory rate, are consistently found to be important predispositions to mortality following discharge. Though the results of this study do not address issues of causality, the recently published Childhood Acute Illness and Nutrition (CHAIN) Network study has carefully built structural equation models to better understand the complex causal pathways related to post-discharge mortality.^3^ These have shown the influence of factors such as adverse caregiver characteristics, high-risk household exposures, and factors related to health seeking. Taken together with the finding that most deaths occur between four to eight weeks following discharge, approaches to improve outcomes must incorporate not merely discharge care, but also changes to how children are managed and monitored during this extended period of risk.

A growing body of research has evaluated interventions to improve post-discharge survival in sub-Saharan Africa. These have focused primarily on malaria, anemia, and general acute illness populations.^48^^,^^50^^,^^66^^,^^69^ Within these disease subgroups, only interventions focusing on post-discharge treatment of malarial anemia have demonstrated improvements in survival and have now been incorporated into the WHO guidance for malaria treatment.^48^^,^^69^^,^^84^ It is recommended that children who are hospitalized due to severe anemia in areas with moderate to high malaria transmission receive intermittent antimalarial prophylaxis after discharge to reduce the risk of readmission and mortality.^85^ However, other interventions in anemia populations have not demonstrated any benefits. One RCT conducted in Uganda and Malawi administered multivitamin multimineral supplements, iron and folate, or co-trimoxazole prophylaxis to children with anemia, but failed to show improvement in survival.^50^ For children recovering from general acute illnesses, a study conducted in this same region investigated a 5-day course of azithromycin administered at hospital discharge, but no significant benefit was observed.^66^ Given the complex causal pathway involved, it may be necessary to adopt a broader interventional strategy that tackles factors at the health system level. This need is especially apparent since the majority of post-discharge deaths occur at home rather than through readmissions.^3^^,^^4^ Thus, improved illness recognition and health seeking within the community context must become essential components of any broad strategy aimed to improve outcomes.

An important barrier to improving care in settings where mortality following discharge is high, is that these settings also suffer from strained and poorly functioning health systems. The development of risk models that can aid health workers to appropriately target children who are most at risk of mortality post-discharge can both improve resource efficiency while also providing a child-centered approach to care.^5^^,^^79^^,^^86^ Robust approaches to the development of parsimonious algorithms utilizing relevant objective clinical, laboratory/biomarker, and socioeconomic data may be a valuable strategy to improve clinical decision-making. Such targeted approaches, combined with intensive community follow-up among the most vulnerable, are likely to have an important impact. Further, these strategies can also leverage the increasingly digitized health landscape to ensure integration of risk prediction with routine care provision.

This study was subject to several important limitations. First, we observed significant heterogeneity across all analyses. While not uncommon in meta-analyses of observational studies, this heterogeneity highlights a significant range in outcomes across settings and timeframes. These findings may be secondary to enrollment heterogeneity (inclusion criteria, enrollment patterns, or underlying prevalence of key risk factors), differences in how children were cared for during hospitalization and follow-up, as well as differences in the definitions of risk factors. Due to the time interval of the analysis spanning several decades, changes in background mortality rates may have also contributed to this heterogeneity. Nevertheless, the lower limits of our confidence intervals rule out any possibility that post-discharge mortality is only of minor concern. Second, although the evidence base has increased significantly, there remain notable gaps within certain disease subgroups, particularly among children admitted with pneumonia or diarrheal disease, where only a small number of studies were identified. To help mitigate the impact of these missing data, our analysis pooled sub-group data and survival curves, along with time-specific mortality estimates derived from reconstructed curves. Such methods increased the amount of available data and further strengthened our results by providing a more robust method for understanding the impacts of time on mortality. Finally, a predominant portion of the data was pertaining to children under-five, while estimates for specific age categories such as neonates and school-aged children remained comparatively limited. There is a crucial need for further research focused specifically on these age groups to better inform age-specific risk factors.

In conclusion, this meta-analysis affirms the high rates of pediatric post-discharge mortality in resource-poor countries. The development of management strategies, particularly focused on at-risk groups such as those with malnutrition or anemia, are urgently required. Global health strategy and investment should therefore include pediatric post-discharge mortality as a key area for research and policy innovation.

## Contributors

Designed the study protocol: MOW, MK, MC.

Developed the search terms: MK, MC.

Screened titles, abstracts, and full texts: MK, MC, MOW.

Data extraction: MK, MC, VN.

Conducted the analysis: VN.

Interpreted the data: MK, VN, MOW.

Drafted the manuscript: MK, MOW.

Critically reviewed the manuscript: MK, VN, MC, JT, NKM, JK, EK, STJ, JMA, NK, MOW.

All authors and had full access to all the data in the study and accept responsibility to submit for publication.

## Data sharing statement

Study data are available upon reasonable request to the corresponding author or through the Pediatric Sepsis CoLab: https://borealisdata.ca/dataverse/Pedi_SepsisCoLab.

## Declaration of interests

The authors declare no competing interests.

## References

[1] UNICEF (2023). https://data.unicef.org/resources/levels-and-trends-%20in-child-mortality/.

[2] Madrid L., Casellas A., Sacoor C. (2019). Postdischarge mortality prediction in sub-Saharan Africa. Pediatrics.

[3] The Childhood Acute Illness and Nutrition (CHAIN) Network (2022). Childhood mortality during and after acute illness in Africa and south Asia: a prospective cohort study. Lancet Glob Health.

[4] Wiens M.O., Bone J.N., Kumbakumba E. (2023). Mortality after hospital discharge among children younger than 5 years admitted with suspected sepsis in Uganda: a prospective, multisite, observational cohort study. Lancet Child Adolesc Health.

[5] Akech S., Kwambai T., Wiens M.O., Chandna A., Berkley J.A., Snow R.W. (2023). Tackling post-discharge mortality in children living in LMICs to reduce child deaths. Lancet Child Adolesc Health.

[6] Wiens M.O., Pawluk S., Kissoon N. (2013). Pediatric post-discharge mortality in resource-poor countries: a systematic review. PLoS One.

[7] Nemetchek B., English L., Kissoon N. (2018). Paediatric postdischarge mortality in developing countries: a systematic review. BMJ Open.

[8] Page M.J., McKenzie J.E., Bossuyt P.M. (2021). The PRISMA 2020 statement: an updated guideline for reporting systematic reviews. BMJ.

[9] Chaudhry M., Knappett M., Nguyen V. (2023). Pediatric post-discharge mortality in resource-poor countries: a protocol for an updated systematic review and meta-analysis. PLoS One.

[10] Campbell S.M. (2020). https://docs.google.com/document/d/1LI5i-I2sDTFX93u6tyrAtic3TSsT-daumtWYwno1pMM/edit#.

[11] Campbell S.M. (2020). https://docs.google.com/document/d/1GKL-8VjDOjp-W5HHu3RkVAXHoQVjLxmNVHy_mE-p-CA/edit#.

[12] Leclercq E., Leeflang M.M.G., van Dalen E.C., Kremer L.C.M. (2013). Validation of search filters for identifying pediatric studies in PubMed. J Pediatr.

[13] Munn Z., Moola S., Lisy K., Riitano D., Tufanaru C. (2015). Methodological guidance for systematic reviews of observational epidemiological studies reporting prevalence and cumulative incidence data. Int J Evid Based Healthc.

[14] United Nations Development Programme (2011). https://hdr.undp.org/system/files/documents/human-development-report-2011-english.human-development-report-2011-english.

[15] United Nations Development Programme (2016). https://sustainabledevelopment.un.org/content/documents/25212016_human_development_report.pdf.

[16] Global Burden of Disease Collaborative Network (2020). https://ghdx.healthdata.org/record/ihme-data/gbd-2019-socio-demographic-index-sdi-1950-2019.

[17] Rohatgi A. (2022). https://automeris.io/WebPlotDigitizer/.

[18] Munn Z., Moola S., Lisy K., Riitano D., Tufanaru C., Aromataris E., Munn Z. (2021). JBI manual for evidence synthesis.

[19] Tierney J.F., Stewart L.A., Ghersi D., Burdett S., Sydes M.R. (2007). Practical methods for incorporating summary time-to-event data into meta-analysis. Trials.

[20] Combescure C., Foucher Y., Jackson D. (2014). Meta-analysis of single-arm survival studies: a distribution-free approach for estimating summary survival curves with random effects. Stat Med.

[21] Guyot P., Ades A., Ouwens M.J., Welton N.J. (2012). Enhanced secondary analysis of survival data: reconstructing the data from published Kaplan-Meier survival curves. BMC Med Res Methodol.

[22] Ashraf H., Alam N.H., Chisti M.J., Salam M.A., Ahmed T., Gyr N. (2012). Observational follow-up study following two cohorts of children with severe pneumonia after discharge from day care clinic/hospital in Dhaka, Bangladesh. BMJ Open.

[23] Bangirana P., Opoka R.O., Boivin M.J. (2014). Severe malarial anemia is associated with longterm neurocognitive impairment. Clin Infect Dis.

[24] Berkley J.A., Ngari M., Thitiri J. (2016). Daily co-trimoxazole prophylaxis to prevent mortality in children with complicated severe acute malnutrition: a multicentre, double-blind, randomised placebo-controlled trial. Lancet Glob Health.

[25] Biai S., Rodrigues A., Gomes M. (2007). Reduced in-hospital mortality after improved management of children under 5 years admitted to hospital with malaria: randomised trial. Br Med J.

[26] Bourdon C., Lelijveld N., Thompson D. (2019). Metabolomics in plasma of Malawian children 7 years after surviving severe acute malnutrition: “ChroSAM” a cohort study. EBioMedicine.

[27] Brim R., Mboma S., Semrud-Clikeman M. (2017). Cognitive outcomes and psychiatric symptoms of retinopathy-positive cerebral malaria: cohort description and baseline results. Am J Trop Med Hyg.

[28] Bwakura-Dangarembizi M., Dumbura C., Ngosa D. (2022). Fat and lean mass predict time to hospital readmission or mortality in children treated for complicated severe acute malnutrition in Zimbabwe and Zambia. Br J Nutr.

[29] Bwakura-Dangarembizi M., Dumbura C., Amadi B. (2022). Recovery of children following hospitalisation for complicated severe acute malnutrition. Matern Child Nutr.

[30] Bwakura-Dangarembizi M., Dumbura C., Amadi B. (2021). Risk factors for postdischarge mortality following hospitalization for severe acute malnutrition in Zimbabwe and Zambia. Am J Clin Nutr.

[31] Chami N., Hau D.K., Masoza T.S. (2019). Very severe anemia and one year mortality outcome after hospitalization in Tanzanian children: a prospective cohort study. PLoS One.

[32] Chapagain R.H., Adhikari S., Shrestha N.J. (2022). Clinicolaboratory profile and treatment outcome within ninety days after discharge during second wave of pediatric COVID-19. J Nepal Health Res Counc.

[33] Chhibber A.V., Hill P.C., Jafali J. (2015). Child mortality after discharge from a health facility following suspected pneumonia, meningitis or septicaemia in rural Gambia: a cohort study. PLoS One.

[34] Chisti M.J., Graham S.M., Duke T. (2014). Post-discharge mortality in children with severe malnutrition and pneumonia in Bangladesh. PLoS One.

[35] Conroy A.L., Hawkes M., Elphinstone R.E. (2016). Acute kidney injury is common in pediatric severe malaria and is associated with increased mortality. Open Forum Infect Dis.

[36] Conroy A.L., Hawkes M., McDonald C.R. (2016). Host biomarkers are associated with response to therapy and long-term mortality in pediatric severe malaria. Open Forum Infect Dis.

[37] Conroy A.L., Opoka R.O., Bangirana P. (2019). Acute kidney injury is associated with impaired cognition and chronic kidney disease in a prospective cohort of children with severe malaria. BMC Med.

[38] Conroy A.L., Hawkes M.T., Elphinstone R. (2018). Chitinase-3-like 1 is a biomarker of acute kidney injury and mortality in paediatric severe malaria NCT01255215 NCT. Malar J.

[39] English L., Kumbakumba E., Larson C.P. (2016). Pediatric out-of-hospital deaths following hospital discharge: a mixed-methods study. Afr Health Sci.

[40] Gonzales G.B., Ngari M.M., Njunge J.M. (2020). Phenotype is sustained during hospital readmissions following treatment for complicated severe malnutrition among Kenyan children: a retrospective cohort study. Matern Child Nutr.

[41] Grenov B., Namusoke H., Lanyero B. (2017). Effect of probiotics on diarrhea in children with severe acute malnutrition: a randomized controlled study in Uganda. J Pediatr Gastroenterol Nutr.

[42] Hamaluba M., van der Pluijm R.W., Weya J. (2021). Arterolane–piperaquine–mefloquine versus arterolane–piperaquine and artemether–lumefantrine in the treatment of uncomplicated *Plasmodium falciparum* malaria in Kenyan children: a single-centre, open-label, randomised, non-inferiority trial. Lancet Infect Dis.

[43] Hau D.K., Chami N., Duncan A. (2018). Post-hospital mortality in children aged 2-12 years in Tanzania: a prospective cohort study. PLoS One.

[44] Hawkes M.T., Conroy A.L., Opoka R.O. (2015). Inhaled nitric oxide as adjunctive therapy for severe malaria: a randomized controlled trial. Malar J.

[45] Hennart P., Beghin D., Bossuyt M. (1987). Long-term follow-up of severe protein-energy malnutrition in Eastern Zaire. J Trop Pediatr.

[46] Islam M.A., Rahman M.M., Mahalanabis D., Rahman A.K. (1996). Death in a diarrhoeal cohort of infants and young children soon after discharge from hospital: risk factors and causes by verbal autopsy. J Trop Pediatr.

[47] Kerac M., Bunn J., Chagaluka G. (2014). Follow-up of post-discharge growth and mortality after treatment for severe acute malnutrition (FuSAM study): a prospective cohort study. PLoS One.

[48] Kwambai T.K., Dhabangi A., Idro R. (2020). Malaria chemoprevention in the postdischarge management of severe anemia. N Engl J Med.

[49] Langfitt J.T., Mcdermott M.P., Brim R. (2019). Neurodevelopmental impairments 1 year after cerebral malaria. Pediatrics.

[50] Maitland K., Olupot-Olupot P., Kiguli S. (2019). Co-trimoxazole or multivitamin multimineral supplement for post-discharge outcomes after severe anaemia in African children: a randomised controlled trial. Lancet Glob Health.

[51] Masoza T.S., Rwezaula R., Msanga D.R. (2022). Prevalence and outcome of HIV infected children admitted in a tertiary hospital in Northern Tanzania. BMC Pediatr.

[52] Moisi J.C., Gatakaa H., Berkley J.A. (2011). Excess child mortality after discharge from hospital in Kilifi, Kenya: a retrospective cohort analysis. Bull World Health Organ.

[53] Mukasa O., Masanja H., DeSavigny D., Schellenberg J. (2021). A cohort study of survival following discharge from hospital in rural Tanzanian children using linked data of admissions with community-based demographic surveillance. Emerg Themes Epidemiol.

[54] Mwangome M., Ngari M., Fegan G. (2017). Diagnostic criteria for severe acute malnutrition among infants aged under 6 mo. Am J Clin Nutr.

[55] Mwene-Batu P., Bisimwa G., Ngaboyeka G. (2020). Follow-up of a historic cohort of children treated for severe acute malnutrition between 1988 and 2007 in Eastern Democratic Republic of Congo. PLoS One.

[56] Namazzi R., Batte A., Opoka R.O. (2022). Acute kidney injury, persistent kidney disease, and post-discharge morbidity and mortality in severe malaria in children: a prospective cohort study. EClinicalMedicine.

[57] Ngari M.M., Fegan G., Mwangome M.K. (2017). Mortality after inpatient treatment for severe pneumonia in children: a cohort study. Paediatr Perinat Epidemiol.

[58] Ngari M.M., Thitiri J., Mwalekwa L. (2018). The impact of rickets on growth and morbidity during recovery among children with complicated severe acute malnutrition in Kenya: a cohort study. Matern Child Nutr.

[59] Nkosi-Gondwe T., Calis J., van Hensbroek M.B., Bates I., Blomberg B., Phiri K.S. (2021). A cohort analysis of survival and outcomes in severely anaemic children with moderate to severe acute malnutrition in Malawi. PLoS One.

[60] Nkosi-Gondwe T., Robberstad B., Mukaka M. (2021). Adherence to community versus facility-based delivery of monthly malaria chemoprevention with dihydroartemisinin-piperaquine for the post-discharge management of severe anemia in Malawian children: a cluster randomized trial. PLoS One.

[61] Olupot-Olupot P., Engoru C., Thompson J. (2014). Phase II trial of standard versus increased transfusion volume in Ugandan children with acute severe anemia. BMC Med.

[62] Opoka R.O., Hamre K.E.S., Brand N., Bangirana P., Idro R., John C.C. (2017). High postdischarge morbidity in Ugandan children with severe malarial anemia or cerebral malaria. J Pediatr Infect Dis Soc.

[63] Opoka R.O., Waiswa A., Harriet N., John C.C., Tumwine J.K., Karamagi C. (2020). Blackwater fever in Ugandan children with severe anemia is associated with poor postdischarge outcomes: a prospective cohort study. Clin Infect Dis.

[64] Ouma B.J., Ssenkusu J.M., Shabani E. (2020). Endothelial activation, acute kidney injury, and cognitive impairment in pediatric severe malaria. Crit Care Med.

[65] Page A.L., Boum Y., Kemigisha E. (2017). Aetiology and outcomes of suspected infections of the central nervous system in children in Mbarara, Uganda. Sci Rep.

[66] Pavlinac P.B., Singa B.O., Tickell K.D. (2021). Azithromycin for the prevention of rehospitalisation and death among Kenyan children being discharged from hospital: a double-blind, placebo-controlled, randomised controlled trial. Lancet Glob Health.

[67] Pavlinac P.B., Singa B., Huang M.L. (2022). Cytomegalovirus viremia predicts postdischarge mortality in Kenyan HIV-exposed uninfected children. J Infect Dis.

[68] Phiri K.S., Calis J.C.J., Faragher B. (2008). Long term outcome of severe anaemia in Malawian children. PLoS One.

[69] Phiri K., Esan M., van Hensbroek M.B. (2012). Intermittent preventive therapy for malaria with monthly artemether-lumefantrine for the post-discharge management of severe anaemia in children aged 4-59 months in southern Malawi: a multicentre, randomised, placebo-controlled trial. Lancet Infect Dis.

[70] Rivera-Correa J., Conroy A.L., Opoka R.O. (2019). Autoantibody levels are associated with acute kidney injury, anemia and post-discharge morbidity and mortality in Ugandan children with severe malaria. Sci Rep.

[71] Roy S.K., Chowdhury M.A., Rahaman M.M. (1983). Excess mortality among children discharged from hospital after treatment for diarrhoea in rural Bangladesh. Br Med J.

[72] Shahrin L., Chisti M.J., Brintz B. (2020). Clinical and laboratory predictors of 30-day mortality in severe acute malnourished children with severe pneumonia. Trop Med Int Health.

[73] Talbert A., Ngari M., Bauni E. (2019). Mortality after inpatient treatment for diarrhea in children: a cohort study. BMC Med.

[74] Tickell K.D., Denno D.M., Saleem A. (2022). Enteric permeability, systemic inflammation, and post-discharge growth among a cohort of hospitalized children in Kenya and Pakistan. J Pediatr Gastroenterol Nutr.

[75] Tomczyk S., McCracken J.P., Contreras C.L. (2019). Factors associated with fatal cases of acute respiratory infection (ARI) among hospitalized patients in Guatemala. BMC Public Health.

[76] Veirum J.E., Sodeman M., Biai S., Hedegård K., Aaby P. (2007). Increased mortality in the year following discharge from a paediatric ward in Bissau, Guinea-Bissau. Acta Paediatr.

[77] Villamor E., Misegades L., Fataki M.R., Mbise R.L., Fawzi W.W. (2005). Child mortality in relation to HIV infection, nutritional status, and socio-economic background. Int J Epidemiol.

[78] West T.E., Goetghebuer T., Milligan P., Mulholland E.K., Weber M.W. (1999). Long-term morbidity and mortality following hypoxaemic lower respiratory tract infection in Gambian children. Bull World Health Organ.

[79] Wiens M.O., Kumbakumba E., Larson C.P. (2015). Postdischarge mortality in children with acute infectious diseases: derivation of postdischarge mortality prediction models. BMJ Open.

[80] Ngari M.M., Obiero C., Mwangome M.K. (2020). Mortality during and following hospital admission among school-aged children: a cohort study. Wellcome Open Res.

[81] Stanton B., Clemens J., Khair T., Shahid N.S. (1986). Follow-up of children discharged from hospital after treatment for diarrhea in urban Bangladesh. Trop Geogr Med.

[82] Zucker J.R., Lackritz E.M., Ruebush T.K. (1996). Childhood mortality during and after hospitalization in western Kenya: effect of malaria treatment regimens. Am J Trop Med Hyg.

[83] Njunge J.M., Gwela A., Kibinge N.K. (2019). Biomarkers of post-discharge mortality among children with complicated severe acute malnutrition. Sci Rep.

[84] World Health Organization (2022). https://www.who.int/news/item/03-06-2022-Updated-WHO-recommendations-for-malaria-chemoprevention-among-children-and-pregnant-women.

[85] World Health Organization (2022). https://www.who.int/news/item/03-06-2022-Updated-WHO-recommendations-for-malaria-chemoprevention-among-children-and-pregnant-women.

[86] Wiens M.O., Kissoon N., Kabakyenga J. (2018). Smart hospital discharges to address a neglected epidemic in sepsis in low- and middle-income countries. JAMA Pediatr.

