# Supplementary material for: Pediatric post-discharge mortality in resource-poor countries: a systematic review and meta-analysis
Source: eClinicalMedicine. 2023 Dec 21;67:102380. doi: 10.1016/j.eclinm.2023.102380 (PMC10776442; doi:10.1016/j.eclinm.2023.102380)
Supplement: Appendix [file mmc1.docx]

Pediatric post-discharge mortality in resource-poor countries: a systematic review and meta-analysis

**Supplementary Appendix**

Contents

[Search Terms: Current Publication 3](#_Toc152090123)

[Table S1. Search Strategy Ovid MEDLINE (January 31, 2023). 3](#_Toc152090124)

[Table S2. Search Strategy Ovid Embase (January 31, 2023). 5](#_Toc152090125)

[Table S3. Search Strategy for EBSCO CINAHL (January 31, 2023). 7](#_Toc152090126)

[Search Terms: 2018 Publication 11](#_Toc152090127)

[Table S4. Search Strategy for Ovid MEDLINE (July 18, 2017). 11](#_Toc152090128)

[Table S5. Search Strategy for Ovid Embase (July 18, 2017). 12](#_Toc152090129)

[Search Terms: 2013 Publication 14](#_Toc152090130)

[Country Eligibility 15](#_Toc152090131)

[S6 Table. Country Classification. 15](#_Toc152090132)

[Study Characteristics 19](#_Toc152090133)

[Table S7. Extended study characteristics summary. 19](#_Toc152090134)

[Risk of Bias Assessment 24](#_Toc152090135)

[Table S8. Summary of risk of bias assessment results. 28](#_Toc152090136)

[Publication Bias Assessment 30](#_Toc152090137)

[Figure S1. Six-month post-discharge mortality funnel plot. 30](#_Toc152090138)

[Figure S2. In-hospital mortality funnel plot 30](#_Toc152090139)

[Supplementary Results 31](#_Toc152090140)

[**Six Month Post-Discharge Mortality Estimates** 31](#_Toc152090141)

[Figure S3. Proportion of six-month post-discharge mortality among studies of general acute illness. 31](#_Toc152090142)

[Figure S4. Proportion of six-month post-discharge mortality among population sub-groups. 32](#_Toc152090143)

[**Twelve Month Post-Discharge Mortality Estimates** 33](#_Toc152090144)

[Table S9. 12-month post-discharge mortality fixed and random effects estimates. 33](#_Toc152090145)

[Figure S5. 12-Month post-discharge mortality among general acute illness. 33](#_Toc152090146)

[Figure S6. 12-Month post-discharge mortality among population sub-groups. 34](#_Toc152090147)

[**Hazard Ratio Pooling** 35](#_Toc152090148)

[Table S10. Fixed and random effects pooled hazard ratios. 35](#_Toc152090149)

[Figure S7. Pooled hazard ratios (random-effects) for post-discharge mortality, adjusted estimates only. 36](#_Toc152090150)

[Figure S8. Pooled hazard ratios (random-effects) for post-discharge mortality, unadjusted estimates only. 37](#_Toc152090151)

[**Survival Curve Analysis** 38](#_Toc152090152)

[Table S11. Median time to death post-discharge among disease sub-groups. 38](#_Toc152090153)

[Table S12. Median time to death post-discharge among age sub-groups. 38](#_Toc152090154)

[Figure S9. Individual study mortality curves (1 minus survival) across all sub-groups. 39](#_Toc152090155)

[Figure S10. Individual study mortality curves (1 minus survival) across age groups. 40](#_Toc152090156)

[**In-Hospital Mortality** 41](#_Toc152090157)

[Table S13. In-hospital mortality fixed and random effects estimates. 41](#_Toc152090158)

[Figure S11. In-hospital mortality random effects estimates among general acute illness. 41](#_Toc152090159)

[Figure S12. In-hospital mortality among general acute illness. 42](#_Toc152090160)

[Figure S13. In-hospital mortality among disease sub-groups. 43](#_Toc152090161)

[Figure S14. Difference between post-discharge in-hospital mortality rates. 44](#_Toc152090162)

[Figure S15. In-hospital versus post-discharge proportion of all deaths. 44](#_Toc152090163)

[Figure S16. Post-discharge mortality versus in-hospital mortality proportion scatterplot for general acute illness studies. 45](#_Toc152090164)

[**Sensitivity Analyses** 46](#_Toc152090165)

[Table S14. 6-month post-discharge mortality fixed and random effects estimates for studies whose recruitment end date was after January 1st 2010. 46](#_Toc152090166)

[Table S15. 6-month post-discharge mortality fixed and random effects estimates with RCTs excluded. 46](#_Toc152090167)

# Search Terms: Current Publication

## Table S1. Search Strategy Ovid MEDLINE (January 31, 2023).

| **No.** | **Search Category** | **Terms** | **Hits** |
| --- | --- | --- | --- |
| 1 | Post-Discharge Mortality | exp hospitalization/ or (hospital*).ti,ab. | 1654409 |
| 2 |  | exp mortality/ or (mortality or death* or fatal* or survival).ti,ab. | 2804270 |
| 3 |  | exp follow-up studies/ or exp longitudinal studies/ or Outcome Assessment, Health Care/ or (follow-up* or long term* or postdischarge or (post adj3 discharge) or after discharge or after hospital* or post hospital*).ti,ab. | 2477343 |
| 4 |  | 1 and 2 and 3 | 123738 |
| 5 | Low and Low-Middle SDI ​​Countries Names and General Developing Country Terms | (afghanistan or benin or burkina faso or burkina fasso or burundi or urundi or central african republic or chad or cote d’ivoire or cote d’ ivoire or cote divoire or cote d ivoire or ivory coast or democratic republic of the congo or democratic republic congo or eritrea or ethiopia or guinea or guinea bissau or haiti or liberia or madagascar or malagasy republic or malawi or nyasaland or mali or mozambique or portuguese east africa or nepal or niger or pakistan or papua new guinea or new guinea or rwanda or ruanda or senegal or sierra leone or solomon islands or solomon or somalia or south sudan or tanzania or the gambia or togo or togolese republic or uganda or yemen).ti,ab,sh,kf. | 318055 |
| 6 |  | (angola or bangladesh or belize or bhutan or bolivia or cambodia or cameroon or cameron or cameroun or cape verde or cabo verde or comoros or comoro islands or iles comores or congo or djibouti or dominican republic or el salvador or eswatini or swaziland or ghana or gold coast or guatemala or honduras or india or kenya* or kiribati or kyrgyzstan or kirghizia or kirgizstan or kyrgyz republic or kirghiz or laos or lao pdr or "lao people's democratic republic" or lesotho or basutoland or maldives or micronesia or federated states of micronesia or marshall islands or mauritania or mongolia or morocco or myanmar or burma or nicaragua or nigeria or north korea or "democratic people’s republic of korea" or republic of korea or palestine or "sao tome and principe" or sudan or tajikistan or tadjikistan or tadzhikistan or tadzhik or timor leste or east timor or tuvalu or vanuatu or new hebrides or venezuela or zambia or zimbabwe).ti,ab,sh,kf. | 436174 |
| 7 |  | (developing countr* or developing nation? or developing population? or developing world or less developed countr* or less developed nation? or less developed population? or less developed world or lesser developed countr* or lesser developed nation? or lesser developed population? or lesser developed world or under developed countr* or under developed nation? or under developed population? or under developed world or underdeveloped countr* or underdeveloped nation? or underdeveloped population? or underdeveloped world or low income countr* or low income nation? or low income population? or lower income countr* or lower income nation? or lower income population? or underserved countr* or underserved nation? or underserved population? or underserved world or under served countr* or under served nation? or under served population? or under served world or deprived countr* or deprived nation? or deprived population? or deprived world or poor countr* or poor nation? or poor population? or poor world or poorer countr* or poorer nation? or poorer population? or poorer world or developing econom* or less developed econom* or lesser developed econom* or under developed econom* or underdeveloped econom* or low income econom* or lower income econom* or low gdp or low gnp or low gross domestic or low gross national or lower gdp or lower gnp or lower gross domestic or lower gross national or third world or lami countr* or LMIC or sub-saharan africa).ti,ab,sh,kf. | 198795 |
| 8 |  | 5 or 6 or 7 | 853673 |
| 9 | Combining Categories | 4 and 8 | 5848 |
| 10 | Exclusions | (address or autobiography or bibliography or biography or case reports or classical article or clinical conference or comment or congress or consensus development conference or consensus development conference, nih or dataset or dictionary or directory or editorial or "expression of concern" or festschrift or government publication or guideline or historical article or interactive tutorial or interview or lecture or legal case or legislation or letter or meta analysis or news or newspaper article or patient education handout or periodical index or personal narrative or portrait or practice guideline or "review" or "scientific integrity review" or "systematic review" or technical report or video-audio media or webcast).pt. | 8070258 |
| 11 |  | (exp animal/ or exp invertebrate/ or animal experiment/ or animal model/ or exp plant/ or exp fungus/) not exp human/ | 5510271 |
| 12 |  | exp Neoplasms/ | 3787944 |
| 13 |  | 9 not (10 or 11 or 12) | 4681 |
| 14 |  | exp adolescent/ or exp child/ or exp infant/ or (infant disease* or childhood disease*).ti,ab,kf. or (adolescen* or babies or baby or boy? or boyfriend or boyhood or child* or girl? or infant* or juvenil* or kid? or minors or minors* or neonat* or neonat* or newborn* or new-born* or paediatric* or peadiatric* or pediatric* or perinat* or preschool* or puber* or pubescen* or school* or teen* or toddler? or underage? or under-age? or youth*).ti,ab,kf. | 4921144 |
| 15 |  | 13 and 14 | 2481 |
| 16 | Limiting | limit 15 to yr="2017-Current" | 1078 |

## Table S2. Search Strategy Ovid Embase (January 31, 2023).

| **No.** | **Search Category** | **Terms** | **Hits** |
| --- | --- | --- | --- |
| 1 | Post-Discharge Mortality | exp hospitalization/ or exp hospital discharge/ or (hospital*).ti,ab. | 2610840 |
| 2 |  | exp mortality/ or exp mortality risk/ or exp mortality rate/ or (mortality or death* or fatal* or survival).ti,ab. | 4147514 |
| 3 |  | exp follow-up/ or exp longitudinal study/ or (follow-up* or long term* or postdischarge or (post adj3 discharge) or after discharge or after hospital*).ti,ab. | 3568407 |
| 4 |  | 1 and 2 and 3 | 217050 |
| 5 | Low and Low-Middle SDI Countries Names and General Developing Country Terms | **(afghanistan** or **benin** or **burkina faso** or burkina fasso or **burundi** or urundi or **central african republic** or **chad** or **cote d’ivoire** or cote d’ ivoire or cote divoire or cote d ivoire or ivory coast or democratic republic of the congo or **democratic republic congo** or **eritrea** or **ethiopia** or **guinea** or **guinea bissau** or **haiti** or **liberia** or **madagascar** or malagasy republic or **malawi** or nyasaland or **mali** or **mozambique** or portuguese east africa or **nepal** or **niger** or **pakistan** or **papua new guinea** or new guinea or **rwanda** or ruanda or **senegal** or **sierra leone** or **solomon islands** or solomon or **somalia** or **south sudan** or **tanzania** or **the gambia** or **togo** or togolese republic or **uganda** or **yemen)**.ti,ab,sh,kf. | 343567 |
| 6 |  | **(angola** or **bangladesh** or **belize** or **bhutan** or **bolivia** or c**ambodia** or **cameroon** or cameron or cameroun or **cape verde**  or cabo verde or **comoros** or comoro islands or iles comores or **congo** or **djibouti** or **dominican republic** or **el salvador** or eswatini or **swaziland** or **ghana** or gold coast or **guatemala** or **honduras** or **india** or **kenya*** or **kiribati** or **kyrgyzstan** or kirghizia or kirgizstan or kyrgyz republic or kirghiz or **laos** or lao pdr or "lao people's democratic republic" or **lesotho** or basutoland or **maldives** or micronesia or **federated states of micronesia** or **marshall islands** or **mauritania** or **mongolia** or **morocco** or **myanmar** or burma or **nicaragua** or **nigeria** or **north korea** or "democratic people’s republic of korea" or republic of korea or **palestine** or **"sao tome and principe"** or **sudan** or **tajikistan** or tadjikistan **or** tadzhikistan or tadzhik or **timor leste** or east timor or **tuvalu** or **vanuatu** or new hebrides or **venezuela** or **zambia** or **zimbabwe)**.ti,ab,sh,kf. | 477930 |
| 7 |  | (developing countr* or developing nation? or developing population? or developing world or less developed countr* or less developed nation? or less developed population? or less developed world or lesser developed countr* or lesser developed nation? or lesser developed population? or lesser developed world or under developed countr* or under developed nation? or under developed population? or under developed world or underdeveloped countr* or underdeveloped nation? or underdeveloped population? or underdeveloped world or low income countr* or low income nation? or low income population? or lower income countr* or lower income nation? or lower income population? or underserved countr* or underserved nation? or underserved population? or underserved world or under served countr* or under served nation? or under served population? or under served world or deprived countr* or deprived nation? or deprived population? or deprived world or poor countr* or poor nation? or poor population? or poor world or poorer countr* or poorer nation? or poorer population? or poorer world or developing econom* or less developed econom* or lesser developed econom* or under developed econom* or underdeveloped econom* or low income econom* or lower income econom* or low gdp or low gnp or low gross domestic or low gross national or lower gdp or lower gnp or lower gross domestic or lower gross national or third world or lami countr*).ti,ab,sh,kf. | 200662 |
| 8 |  | 5 or 6 or 7 | 927161 |
| 9 | Combining Categories | 4 and 8 | 8315 |
| 10 | Exclusions | (books or chapter or conference abstract or "conference review" or editorial or erratum or letter or note or "review" or short survey or tombstone).pt. | 11314957 |
| 11 |  | (exp animal/ or exp invertebrate/ or nonhuman/ or animal experiment/ or animal tissue/ or animal model/ or exp plant/ or exp fungus/) not (exp human/ or human tissue/) | 7582441 |
| 12 |  | exp Neoplasms/ | 5354402 |
| 13 |  | 9 not (10 or 11 or 12) | 4334 |
| 14 | Limiting | exp adolescence/ or exp adolescent/ or exp child/ or exp childhood disease/ or exp infant disease/ or (adolescen* or babies or baby or boy? or boyfriend or boyhood or child or child* or child*3 or children* or girl? or infant* or juvenil* or juvenile* or kid? or minors or minors* or neonat* or neonat* or newborn* or new-born* or paediatric* or peadiatric* or pediatric* or perinat* or preschool* or puber* or pubescen* or school or school child* or school* or schoolchild* or schoolchild* or teen* or toddler? or underage? or under-age? or youth*).ti,ab,kf,hw. | 6137389 |
| 15 |  | 13 and 14 | 2477 |
| 16 |  | limit 15 to yr="2017-Current" | 1262 |

## Table S3. Search Strategy for EBSCO CINAHL (January 31, 2023).

| ID | Search | Hits |
| --- | --- | --- |
| S14 | S11 OR S12 Limiters - Published Date: 20170101-20221231 | 609 |
| S13 | S11 OR S12 | 1199 |
| S12 | S9 AND S10 | 852 |
| S11 | S7 AND S8 Limiters - Age Groups: Infant: 1-23 months, Child, Preschool: 2-5 years, Child: 6-12 years, Adolescent: 13-18 years | 983 |
| S10 | TI (pediatric* OR paediatric* OR child* OR baby OR babies OR infan* OR toddler* OR preteen* OR (pre W1 teen*) OR preadolescen* OR (pre W1 adolescen*) OR youth* OR youngster* OR boy* OR girl* OR juvenile*) OR AB (pediatric* OR paediatric* OR child* OR baby OR babies OR infan* OR toddler* OR preschool* OR preteen* OR (pre W1 teen*) OR preadolescen* OR (pre W1 adolescen*) OR youth* OR youngster* OR boy* OR girl* OR juvenile*) OR JN (pediatric* OR paediatric* OR child* OR baby OR babies OR infan* OR toddler* OR preteen* OR (pre W1 teen*) OR preadolescen* OR (pre W1 adolescen*) OR youth* OR youngster* OR boy* OR girl* OR juvenile*) | 897486 |
| S9 | S7 AND S8 | 2473 |
| S8 | S4 OR S5 OR S6 | 211967 |
| S7 | S1 AND S2 AND S3 | 47752 |
| S6 | (MH "Developing Countries") OR (TI (developing countr* or developing nation? or developing population? or developing world or less developed countr* or less developed nation? or less developed population? or less developed world or lesser developed countr* or lesser developed nation? or lesser developed population? or lesser developed world or under developed countr* or under developed nation? or under developed population? or under developed world or underdeveloped countr* or underdeveloped nation? or underdeveloped population? or underdeveloped world or low income countr* or low income nation? or low income population? or lower income countr* or lower income nation? or lower income population? or underserved countr* or underserved nation? or underserved population? or underserved world or under served countr* or under served nation? or under served population? or under served world or deprived countr* or deprived nation? or deprived population? or deprived world or poor countr* or poor nation? or poor population? or poor world or poorer countr* or poorer nation? or poorer population? or poorer world or developing econom* or less developed econom* or lesser developed econom* or under developed econom* or underdeveloped econom* or low income econom* or lower income econom* or low gdp or low gnp or low gross domestic or low gross national or lower gdp or lower gnp or lower gross domestic or lower gross national or third world or lami countr*)) OR (AB (developing countr* or developing nation? or developing population? or developing world or less developed countr* or less developed nation? or less developed population? or less developed world or lesser developed countr* or lesser developed nation? or lesser developed population? or lesser developed world or under developed countr* or under developed nation? or under developed population? or under developed world or underdeveloped countr* or underdeveloped nation? or underdeveloped population? or underdeveloped world or low income countr* or low income nation? or low income population? or lower income countr* or lower income nation? or lower income population? or underserved countr* or underserved nation? or underserved population? or underserved world or under served countr* or under served nation? or under served population? or under served world or deprived countr* or deprived nation? or deprived population? or deprived world or poor countr* or poor nation? or poor population? or poor world or poorer countr* or poorer nation? or poorer population? or poorer world or developing econom* or less developed econom* or lesser developed econom* or under developed econom* or underdeveloped econom* or low income econom* or lower income econom* or low gdp or low gnp or low gross domestic or low gross national or lower gdp or lower gnp or lower gross domestic or lower gross national or third world or lami countr*)) OR (MW (developing countr* or developing nation? or developing population? or developing world or less developed countr* or less developed nation? or less developed population? or less developed world or lesser developed countr* or lesser developed nation? or lesser developed population? or lesser developed world or under developed countr* or under developed nation? or under developed population? or under developed world or underdeveloped countr* or underdeveloped nation? or underdeveloped population? or underdeveloped world or low income countr* or low income nation? or low income population? or lower income countr* or lower income nation? or lower income population? or underserved countr* or underserved nation? or underserved population? or underserved world or under served countr* or under served nation? or under served population? or under served world or deprived countr* or deprived nation? or deprived population? or deprived world or poor countr* or poor nation? or poor population? or poor world or poorer countr* or poorer nation? or poorer population? or poorer world or developing econom* or less developed econom* or lesser developed econom* or under developed econom* or underdeveloped econom* or low income econom* or lower income econom* or low gdp or low gnp or low gross domestic or low gross national or lower gdp or lower gnp or lower gross domestic or lower gross national or third world or lami countr*)) | 61566 |
| S5 | (TI (angola or bangladesh or belize or bhutan or bolivia or cambodia or cameroon or cameron or cameroun or cape verde or cabo verde or comoros or comoro islands or iles comores or congo or djibouti or dominican republic or el salvador or eswatini or swaziland or ghana or gold coast or guatemala or honduras or india or kenya* or kiribati or kyrgyzstan or kirghizia or kirgizstan or kyrgyz republic or kirghiz or laos or lao pdr or "lao people's democratic republic" or lesotho or basutoland or maldives or micronesia or federated states of micronesia or marshall islands or mauritania or mongolia or morocco or myanmar or burma or nicaragua or nigeria or north korea or "democratic people’s republic of korea" or republic of korea or palestine or "sao tome and principe" or sudan or tajikistan or tadjikistan or tadzhikistan or tadzhik or timor leste or east timor or tuvalu or vanuatu or new hebrides or venezuela or zambia or Zimbabwe)) OR (AB (angola or bangladesh or belize or bhutan or bolivia or cambodia or cameroon or cameron or cameroun or cape verde or cabo verde or comoros or comoro islands or iles comores or congo or djibouti or dominican republic or el salvador or eswatini or swaziland or ghana or gold coast or guatemala or honduras or india or kenya* or kiribati or kyrgyzstan or kirghizia or kirgizstan or kyrgyz republic or kirghiz or laos or lao pdr or "lao people's democratic republic" or lesotho or basutoland or maldives or micronesia or federated states of micronesia or marshall islands or mauritania or mongolia or morocco or myanmar or burma or nicaragua or nigeria or north korea or "democratic people’s republic of korea" or republic of korea or palestine or "sao tome and principe" or sudan or tajikistan or tadjikistan or tadzhikistan or tadzhik or timor leste or east timor or tuvalu or vanuatu or new hebrides or venezuela or zambia or Zimbabwe)) OR (MW (angola or bangladesh or belize or bhutan or bolivia or cambodia or cameroon or cameron or cameroun or cape verde or cabo verde or comoros or comoro islands or iles comores or congo or djibouti or dominican republic or el salvador or eswatini or swaziland or ghana or gold coast or guatemala or honduras or india or kenya* or kiribati or kyrgyzstan or kirghizia or kirgizstan or kyrgyz republic or kirghiz or laos or lao pdr or "lao people's democratic republic" or lesotho or basutoland or maldives or micronesia or federated states of micronesia or marshall islands or mauritania or mongolia or morocco or myanmar or burma or nicaragua or nigeria or north korea or "democratic people’s republic of korea" or republic of korea or palestine or "sao tome and principe" or sudan or tajikistan or tadjikistan or tadzhikistan or tadzhik or timor leste or east timor or tuvalu or vanuatu or new hebrides or venezuela or zambia or Zimbabwe)) | 112976 |
| S4 | (TI (afghanistan or benin or burkina faso or burkina fasso or burundi or urundi or central african republic or chad or cote d’ivoire or cote d’ ivoire or cote divoire or cote d ivoire or ivory coast or democratic republic of the congo or democratic republic congo or eritrea or ethiopia or guinea or guinea bissau or haiti or liberia or madagascar or malagasy republic or malawi or nyasaland or mali or mozambique or portuguese east africa or nepal or niger or pakistan or papua new guinea or new guinea or rwanda or ruanda or senegal or sierra leone or solomon islands or solomon or somalia or south sudan or tanzania or the gambia or togo or togolese republic or uganda or yemen)) OR (AB (afghanistan or benin or burkina faso or burkina fasso or burundi or urundi or central african republic or chad or cote d’ivoire or cote d’ ivoire or cote divoire or cote d ivoire or ivory coast or democratic republic of the congo or democratic republic congo or eritrea or ethiopia or guinea or guinea bissau or haiti or liberia or madagascar or malagasy republic or malawi or nyasaland or mali or mozambique or portuguese east africa or nepal or niger or pakistan or papua new guinea or new guinea or rwanda or ruanda or senegal or sierra leone or solomon islands or solomon or somalia or south sudan or tanzania or the gambia or togo or togolese republic or uganda or yemen)) OR (MW (afghanistan or benin or burkina faso or burkina fasso or burundi or urundi or central african republic or chad or cote d’ivoire or cote d’ ivoire or cote divoire or cote d ivoire or ivory coast or democratic republic of the congo or democratic republic congo or eritrea or ethiopia or guinea or guinea bissau or haiti or liberia or madagascar or malagasy republic or malawi or nyasaland or mali or mozambique or portuguese east africa or nepal or niger or pakistan or papua new guinea or new guinea or rwanda or ruanda or senegal or sierra leone or solomon islands or solomon or somalia or south sudan or tanzania or the gambia or togo or togolese republic or uganda or yemen)) | 61645 |
| S3 | (MH "Prospective Studies+") OR (MH "After Care") OR (TI (follow up* OR long term* OR postdischarge OR post discharge OR after discharge OR after hospital*)) OR (AB (follow up* OR long term* OR postdischarge OR post discharge OR after discharge OR after hospital*)) | 904253 |
| S2 | (MH "Mortality+") OR (TI (mortality OR death* OR fatal* OR survival)) OR (AB (mortality OR death* OR fatal* OR survival)) | 583368 |
| S1 | (MH "hospitalization") OR (MH "Infant, Hospitalized") OR (MH "Adolescent, Hospitalized") OR (MH "Child, Hospitalized") OR (TI (hospital*)) OR (AB (hospital*)) | 573478 |

# Search Terms: 2018 Publication

## Table S4. Search Strategy for Ovid MEDLINE (July 18, 2017).

| **No.** | **Search Category** | **Terms** | **Hits** |
| --- | --- | --- | --- |
| 1 | Post-Discharge Mortality | Exp Follow-Up Studies/ or exp Hospitalization/ or exp Longitudinal Studies/ or (postadjdischarge or after discharge or longadjterm outcome* or longadjterm followadjup or longadjterm mortality or followadjup stud*).ti,ab. | 880860 |
| 2 |  | exp Mortality/ or (mortality or postadjdischarge mortality or postadjdischarge death or postadjdischarge fatal* or postadjhospital*adjmortality or postadjhospital* fatal* or postadjhospital* death or longadjterm mortality or postadjhospital mortality).ti,ab. | 770469 |
| 3 |  | 1 and 2 | 128056 |
| 4 | Developing Countries | exp Africa/ or exp Haiti/ or exp Afghanistan/ or exp Yemen/ or exp Melanesia/ or exp Syria/ or exp Developing Countries/ or (Africa* or Haiti or Afghanistan or Yemen or Melanesia or Solomon Island* or Papua New Guinea or Syria*).ti,ab. | 401121 |
| 5 |  | (lowadjresource countr* or resource poor countr* or developing countr*).ti,ab. | 44097 |
| 6 |  | exp Bangladesh/ or exp Myanmar/ or exp Pakistan/ or (Bangladesh or Myanmar or Burma or Pakistan).ti,ab. | 30050 |
| 7 |  | 4 or 5 or 6 | 445820 |
| 8 | Combining Categories | 3 and 7 | 4093 |
| 9 | Exclusions | ((comment or editorial or meta-analysis or practice-guideline or review or letter or journal correspondence or posters or News or Newspaper article or meeting abstracts or lectures or interview or historical article or handbooks or guidelines or guidebooks or essays or editorial or database or comment or clinical conference or catalogs) not "randomized controlled trial").pt. | 4255011 |
| 10 |  | Animals/ | 6167864 |
| 11 |  | 8 not (9 or 10) | 3836 |
| 12 | Limiting | limit 11 to ("infant (1 to 23 months)" or "preschool child (2 to 5 years)" or "child (6 to 12 years)") | 1710 |
| 13 |  | limit 12 to yr="2012 -Current" | 601 |
| 14 |  | limit 13 to english | 577 |

## Table S5. Search Strategy for Ovid Embase (July 18, 2017).

| **No.** | **Search Category** | **Terms** | **Hits** |
| --- | --- | --- | --- |
| 1 | Post-Discharge Mortality | exp follow up/ or exp hospitalization/ or exp longitudinal study/ or (postadjdischarge or after discharge or longadjterm outcome* or longadjterm followadjup or longadjterm mortality or followadjup stud*).ti,ab | 1516787 |
| 2 |  | exp mortality rate/ or exp mortality/ or exp mortality risk/ or exp childhood mortality/ or exp childhood death/ or (mortality or postadjdischarge mortality or postadjdischarge death or postadjdischarge fatal* or postadjhospital*adjmortality or postadjhospital* fatal* or postadjhospital* death or longadjterm mortality or postadjhospital mortality).ti,ab. | 1193617 |
| 3 |  | 1 and 2 | 220868 |
| 4 | Developing Countries | exp Africa/ or exp Haiti/ or exp Afghanistan/ or exp Yemen/ or exp Solomon Islands/ or exp Syrian Arab Republic/ or exp Papua New Guinea/ or (Africa* or Haiti or Afghanistan or Yemen or Melanesia or Solomon Island* or Papua New Guinea or Syria*).ti,ab. | 440592 |
| 5 |  | exp developing country/ or (lowadjresource countr* or resourceadjpoor countr* or developing countr*).ti,ab. | 120202 |
| 6 |  | exp Bangladesh/ or exp Myanmar/ or exp Pakistan/ or (Bangladesh or Myanmar or Burma or Pakistan).ti,ab. | 42892 |
| 7 |  | 4 or 5 or 6 | 568957 |
| 8 | Combining Categories | 3 and 7 | 7569 |
| 9 | Exclusions | ((comment or editorial or meta-analysis or practice-guideline or review or letter or journal correspondence or posters or News or Newspaper article or meeting abstracts or lectures or interview or historical article or handbooks or guidelines or guidebooks or essays or editorial or database or comment or clinical conference or catalogs) not "randomized controlled trial").pt. | 3811629 |
| 10 |  | Animals/ | 1649863 |
| 11 |  | 8 not (9 or 10) | 7019 |
| 12 | Limiting | limit 11 to (infant <to one year> or child <unspecified age> or preschool child <1 to 6 years> or school child <7 to 12 years>) | 1804 |
| 13 |  | limit 12 to yr="2012 -Current" | 909 |
| 14 |  | limit 13 to english | 898 |

# Search Terms: 2013 Publication

We conducted a systematic computerized search from the inception date (1946 in MEDLINE and 1974 in EMBASE) to October, 2012 to identify all potentially eligible studies. We applied the following algorithm in both medical subject heading (MeSH) and free text words. In MEDLINE, the MeSH terms “follow-up studies,” “hospitalization,” OR “longitudinal studies” were combined with “developing countries,” “Africa,” “Bangladesh,” “Haiti,” “Afghanistan,” “Yemen,” “Papua New Guinea,” “Myanmar,” “Pakistan,” OR “Solomon Islands.” MeSH terms were exploded where appropriate. The MeSH term “Africa” included the names of all African countries when exploded. Free text words including “post-discharge mortality” and “long-term outcomes” were also used to increase capture of relevant articles. In EMBASE, the MeSH terms “follow-up,” “hospitalization,” OR “longitudinal study” were combined with “developing country,” “Bangladesh,” “Haiti,” “Afghanistan,” “Yemen,” “Papua New Guinea,” “Burma,” “Pakistan,” “Solomon Islands” OR “Melanesia” AND “Pediatrics.” Free text word “Burma” was also included in the search as this was not a MeSH term. Google Scholar™ was also searched and references of relevant publications were reviewed to identify any articles not captured during initial search.

# Country Eligibility

## S6 Table. Country Classification.

| **Countries Classified as Low-HDI (2011)^1^** | **Countries Classified as Low-HDI (2016)^2^** | **Countries Classified as Low and Low-Middle SDI (2019)^3^** |
| --- | --- | --- |
| Solomon Islands | Swaziland | Somalia |
| Kenya | Syrian Arab Republic | Niger |
| São Tomé and Príncipe | Angola | Chad |
| Pakistan | Tanzania, United Republic of | Burkina Faso |
| Bangladesh | Nigeria | Mali |
| Timor-Leste | Cameroon | Central African Republic |
| Angola | Papua New Guinea | Burundi |
| Yanmar | Zimbabwe | Mozambique |
| Cameroon | Solomon Islands | Guinea |
| Madagascar | Mauritania | Afghanistan |
| United Republic of Tanzania | Madagascar | Ethiopia |
| Papua New Guinea | Rwanda | Sierra Leone |
| Yemen | Comoros | Benin |
| Senegal | Lesotho | Guinea-Bissau |
| Nigeria | Senegal | South Sudan |
| Nepal | Haiti | Liberia |
| Haiti | Uganda | Democratic Republic of the Congo |
| Mauritania | Sudan | Malawi |
| Lesotho | Togo | Senegal |
| Uganda | Benin | Papua New Guinea |
| Togo | Yemen | Eritrea |
| Comoros | Afghanistan | Madagascar |
| Zambia | Malawi | The Gambia |
| Djibouti | Côte d’Ivoire | Uganda |
| Rwanda | Djibouti | Solomon Islands |
| Benin | Gambia | Côte d'Ivoire |
| Gambia | Ethiopia | Yemen |
| Sudan | Mali | Togo |
| Côte d'Ivoire | Democratic Republic of the Congo | Nepal |
| Malawi | Liberia | Tanzania |
| Afghanistan | Guinea-Bissau | Rwanda |
| Zimbabwe | Eritrea | Haiti |
| Ethiopia | Sierra Leone | Pakistan |
| Mali | Mozambique | Bhutan |
| Guinea-Bissau | South Sudan | Comoros |
| Eritrea | Guinea | Djibouti |
| Guinea | Burundi | Cambodia |
| Central African Republic | Burkina Faso | Angola |
| Sierra Leone | Chad | Zimbabwe |
| Burkina Faso | Niger | Bangladesh |
| Liberia | Central African Republic | Vanuatu |
| Chad |  | Laos |
| Mozambique |  | Cameroon |
| Burundi |  | Honduras |
| Niger |  | Mauritania |
| Democratic Republic of the Congo |  | São Tomé and PrÍncipe |
|  |  | Zambia |
|  |  | Lesotho |
|  |  | Kenya |
|  |  | Timor-Leste |
|  |  | Sudan |
|  |  | Nigeria |
|  |  | Nicaragua |
|  |  | Myanmar |
|  |  | Cape Verde |
|  |  | Guatemala |
|  |  | Kiribati |
|  |  | Tajikistan |
|  |  | Marshall Islands |
|  |  | Morocco |
|  |  | Ghana |
|  |  | North Korea |
|  |  | Maldives |
|  |  | Bolivia |
|  |  | India |
|  |  | Congo |
|  |  | El Salvador |
|  |  | eSwatini (formerly Swaziland) |
|  |  | Federated States of Micronesia (Micronesia) |
|  |  | Palestine |
|  |  | Tuvalu |
|  |  | Dominican Republic |
|  |  | Kyrgyzstan |
|  |  | Belize |
|  |  | Mongolia |
|  |  | Venezuela |

**References**

1. United Nations Development Programme. Human development report 2011: sustainability and equity: a better future for all. 2011. https://hdr.undp.org/system/files/documents/human-development-report-2011-english.human-development-report-2011-english (accessed September 10, 2022)
2. United Nations Development Programme. Human development report 2016: human development for everyone. 2016. https://sustainabledevelopment.un.org/content/documents/25212016_human_development_report.pdf (accessed September 10, 2022)
3. Global Burden of Disease Collaborative Network. Global Burden of Disease Study 2019 (GBD 2019) Socio-Demographic Index (SDI) 1950–2019. 2020. https://ghdx.healthdata.org/record/ihme-data/gbd-2019-socio-demographic-index-sdi-1950-2019 (accessed Sep 10, 2022).

# Study Characteristics

## Table S7. Extended study characteristics summary.

|  | **Years of Study** | **Country** | **Study Design** | **Facility Type** | **Study Population**  **Enrolled** | **Population Subgroups Represented** | **Children Enrolled, N** | **Age Range** | **Age estimate (months): mean (SD) or median (IQR)** | **Female proportion (%)** | **Duration of Follow-up** | **Lost to Follow-up Rate (%)** |
| --- | --- | --- | --- | --- | --- | --- | --- | --- | --- | --- | --- | --- |
| Ashraf et al., 2012 | 2006-2008 | Bangladesh | Prospective Cohort Study | Hospital | Severe pneumonia | Respiratory infections | 180 | 2-59 months | 7.3(6.8) | 34.44 | 3 months | 6.36 |
| Berkley et al., 2016 | 2009-2013 | Kenya | RCT | Hospital | Severe acute malnutrition | Severe malnutrition | 1781 | 60 days-59 months | 11 (7-16) | 49.21 | 12 months | 3.26 |
| Biai et al., 2007 | 2004-2006 | Guinea-Bissau | RCT | Hospital | Malaria | Malaria | 951 | 3-60 months | 24(NA) | 44.16 | 28 days | 4.41 |
| Brim et al., 2017 | 2012-2014 | Malawi | Prospective Case-Control | Hospital | Cerebral malaria | Malaria | 258 | >12 months | NA | 49.32 | 12 months | 18.8 |
| Bwakura‐Dangarembizi et al., 2021a | 2016-2018 | Zambia, Zimbabwe | Prospective Cohort Study | Hospital | Severe acute malnutrition | Severe malnutrition | 755 | 0-60 months | 18.2(13.6-22.6) | 40.40 | 52 weeks | 3.70 |
| CHAIN Network, 2022 | 2016-2019 | Bangladesh, Burkina Faso, Kenya, Malawi, Pakistan, Uganda | Prospective Cohort Study | Hospital | Acute illness | General acute illness | 3101 | 2-23 months | 11(6.8-16) | 43.34 | 180 days | 4.04 |
| Chapagain et al., 2022 | 2021 | Nepal | Retrospective Chart Review with Prospective Follow-up | Hospital | COVID-19 | Other | 156 | 0-14 years | 15(63) | 45.51 | 90 days | 0 |
| Chhibber et al., 2015 | 2008-2012 | Gambia | Prospective Cohort Study | Health Centre | Pneumonia, sepsis, or meningitis | General acute illness | 3952 | 2-59 months | NA | NA | 180 days | NA |
| Chisti et al., 2014 | 2011-2012 | Bangladesh | Prospective Cohort Study | Hospital | Severe malnutrition and radiological pneumonia | Severe malnutrition, respiratory infections | 405 | 0-59 months | 10(5-18) | 44.17 | 12 weeks | 14.67 |
| Grenov et al., 2017 | 2012-2013 | Uganda | RCT | Hospital | Severe acute malnutrition | Severe malnutrition | 400 | 6-59 months | 17(NA) | 42.50 | 8 to 12 weeks | 9.17 |
| Hamaluba et al., 2021 | 2018-2019 | Kenya | RCT | Hospital | Uncomplicated, non-severe malaria | Malaria | 217 | 2-12 years | 85.2(55.2-115.2) | 48.39 | 42 days | 0.46 |
| Hau et al., 2018 | 2014 | Tanzania | Prospective Cohort Study | Hospital | All admissions | General acute illness | 506 | 2-12 years | 54.5(32.5) | 42.29 | 12 months | 20.34 |
| Hawkes et al., 2015 | 2011 | Uganda | RCT | Hospital | Severe malaria | Malaria | 180 | 1-10 years | 24(12-36) | 43.33 | 6 months | 10.96 |
| Hennart et al., 1987 | 1970 | Zaire (Democratic Republic of the Congo) | Prospective Cohort Study | Hospital | Severe protein-energy malnutrition | Severe malnutrition | 171 | 0-6+ years | 46(NA) | NA | 5 years | NA |
| Islam et al., 1996 | 1991-1992 | Bangladesh | Prospective Cohort Study | Hospital | Diarrhea | Diarrhea | 500 | 1-23 months | NA | 39.11 | 12 weeks | 20.2 |
| Kerac et al., 2014 | 2006-2007 | Malawi | Prospective Cohort Study | Hospital | Malnutrition | Severe malnutrition | 1024 | 5-168 months | 21.5(15-32) | 46.97 | 1 year | 17.18 |
| Kwambai et al., 2020 | 2016-2018 | Kenya, Uganda | RCT | Hospital | Severe malaria | Malaria | 525 | 0-5 years | 26.3(14.8) | 49.52 | 26 weeks | 4.95 |
| Madrid et al., 2019 | 2000-2016 | Mozambique | Retrospective Cohort Study | Hospital | All admissions | General acute illness | 18023 | 0-15 years | NA | 45.14 | 90 days | NA |
| Maitland et al., 2019 | 2014-2017 | Malawi, Uganda | RCT | Hospital | Severe anemia | Severe anemia | 3983 | 2 months-12 years | 35(17-61) | 43.38 | 180 days | 4.08 |
| Masoza et al., 2022 | 2014-2015 | Tanzania | Cross-sectional study with prospective follow-up | Hospital | All admissions | General acute illness | 525 | 1 month-12 years | 28(15-56) | 39.81 | 3 months | 2.55 |
| Moisi et al. (2011) | 2004-2008 | Kenya | Retrospective Cohort Study | Hospital | All admissions | General acute illness | 10277 | 0-15 years | NA | NA | 1 year | NA |
| Mukasa et al., 2021 | 2003-2007 | Tanzania | Retrospective Cohort Study | Hospital | All admissions | General acute illness | 861 | <5 years | 27(NA) | 50.07 | 6 months | 0 |
| Mwangome et al., 2017 | 2007-2014 | Kenya | Prospective Cohort Study | Hospital | All admissions | General acute illness | 2882 | 1-6 months | 3(1.7-4.5) | 39.97 | 1 year | 3.44 |
| Mwene-Batu et al., 2020 | 1988-2007 | Democratic Republic of Congo | Retrospective Cohort Study | Hospital | Severe acute malnutrition | Severe malnutrition | 1981 | 0-144 months | 41(NA) | 42.50 | Unspecified, long-term follow-up | 32.61 |
| Namazzi et al., 2022 | 2014-2017 | Uganda | Prospective Cohort Study | Hospital | Severe malaria | Malaria | 598 | 6 months-4 years | 25.2(10.8) | 44.10 | 12 months | 6.13 |
| Ngari et al., 2017 | 2007-2012 | Kenya | Prospective Cohort Study | Hospital | Admitted with or without severe pneumonia | Respiratory infections, general acute illness, HIV | 7731 | 1-59 months | NA | 43.47 | 12 months | 1.96 |
| Ngari et al., 2020 | 2007-2016 | Kenya | Retrospective Cohort Study | Hospital | All admissions | General acute illness, HIV | 3196 | 60-155 months | 92(74-116) | 42.82 | 12 months | 2.19 |
| Nkosi-Gondwe et al., 2021b | 2016-2018 | Malawi | RCT | Hospital | Severe anemia | Severe anemia | 375 | 0-5 years | 29(19-39) | 43.47 | 15 weeks | 3.73 |
| Olupot-Olupot et al., 2014 | 2011-2012 | Uganda | RCT | Hospital | Severe anemia | Severe anemia | 160 | 60 days-12 years | 36(13-53) | 50.63 | 28 days | 0 |
| Opoka et al., 2019 | 2016-2018 | Uganda | Prospective Cohort Study | Hospital | Severe anemia | Severe anemia | 282 | 0-5 years | NA | 36.92 | 6 months | 1.06 |
| Ouma et al., 2020 | 2008-2013 | Uganda | Prospective Cohort Study | Hospital | Cerebral malaria and severe malarial anemia | Malaria, severe anemia | 502 | 18 months-5 years | NA | 35.49 | 24 months | 4.07 |
| Page et al., 2017 | 2009-2012 | Uganda | Prospective Cohort Study | Hospital | Suspected infections of the central nervous system | Other | 459 | 2 months-12 years | 30(11-60) | 36.60 | 6 months | 13.30 |
| Pavlinac et al., 2021 | 2016-2019 | Kenya | RCT | Hospital | All admissions | General acute illness | 1400 | 1-59 months | 18(9-32) | 40.77 | 6 months | NA |
| Phiri et al., 2008 | 2002-2004 | Malawi | "Longitudinal case-control study" | Hospital | Severe anemia | Severe anemia | 758 | 0-5 years | NA | 50.66 | 18 months | 17.85 |
| Phiri et al., 2012 | 2006-2009 | Malawi | RCT | Hospital | Severe malarial anemia | Malaria, severe anemia | 1414 | 4-59 months | 23.9(13.4) | 51.63 | 6 months | 4.95 |
| Roy et al., 1983 | 1979-1980 | Bangladesh | Prospective Cohort Study | Health Centre | Diarrhea | Diarrhea | 551 | 3-36 months | NA | NA | 12 months | NA |
| Shahrin et al., 2020 | 2015-2017 | Bangladesh | Prospective Cohort Study | Hospital | Admitted for diarrhea and had both severe pneumonia and severe acute malnutrition | Diarrhea, respiratory infections, severe malnutrition | 191 | 0-59 months | 8(5-12) | 38.74 | 30 days | 4.14 |
| Stanton et al., 1986 | 1983 | Bangladesh | Retrospective Cohort Study | Hospital | Diarrhea | Diarrhea | 112 | 24-72 months | NA | 27.03 | 4 to 5 months | 6.76 |
| Talbert et al., 2019 | 2007-2015 | Kenya | Retrospective Cohort Study | Hospital | Admitted with or without diarrhea | General acute illness, diarrhea, pneumonia | 17442 | 2-59 months | 18(9-32) | 43.10 | 1 year | 5.57 |
| Tomczyk et al., 2019 | 2007-2013 | Guatemala | Prospective Cohort Study | Hospital | Acute respiratory illness | Respiratory infections | 4109 | 0-2 years | NA | 41.96 | 6 weeks | NA |
| Veirum et al., 2007 | 1991-1996 | Guinea-Bissau | Prospective Cohort Study | Hospital | All admissions | General acute illness | 3373 | 0-6 years | NA |  | 365 days | NA |
| Villamor et al., 2005 | 1993-1997 | Tanzania | Prospective Cohort Study | Hospital | Pneumonia | Respiratory infections | 687 | 6-60 months | 17.6(12.1) | 45.76 | 2 years | 11.41 |
| West et al., 1999 | 1991-1994 | Gambia | Case-Control | Hospital | Acute lower respiratory tract infections | Respiratory infections | 190 | 0-5 years | 9.7(NA) | 43.22 | 2 to 5 years | 37.89 |
| Wiens et al., 2015 | 2012-2013 | Uganda | Prospective Cohort Study | Hospital | Proven or suspected infection | General acute illness, diarrhea, malaria, severe malnutrition, respiratory infections, HIV | 1307 | 6 months-5 years | 8.1(10.8-34.6) | 45.09 | 6 months | 1.69 |
| Wiens et al., 2023 | 2017-2020 | Uganda | Prospective Cohort Study | Hospital | Suspected sepsis | General acute illness, severe anemia, diarrhea, malaria, severe malnutrition, respiratory infections, HIV | 6545 | 0-60 months | 9.6(2.4-19.2) | 47.35 | 6 months | 1.91 |
| Zucker et al., 1996 | 1991 | Kenya | Case-Control | Hospital | Anemia | Severe anemia | 584 | 0-5 years | 12.6(15.7) | 50.00 | 8 weeks | NA |

NA=Not available; SD=Standard deviation; IQR=Interquartile range

# Risk of Bias Assessment

**Critical Appraisal Checklist:**

| **Item** | **Low Risk of Bias** | **Moderate Risk of Bias** | **High Risk of Bias/ Not mentioned/ Unclear** |
| --- | --- | --- | --- |
| 1. Was the sample frame appropriate to address the target population? | □ | □ | □ |
| 1. Were study participants recruited in an appropriate way? | □ | □ | □ |
| 1. Were the study subjects and the setting described in detail? | □ | □ | □ |
| 1. Were valid methods used for the identification of the condition and risk factors? | □ | □ | □ |
| 1. Were the condition and risk factors measured in a standard, reliable way for all participants? | □ | □ | □ |
| 1. Was there appropriate statistical analysis? | □ | □ | □ |
| 1. Was the response rate adequate, and if not, was the low response rate managed appropriately? | □ | □ | □ |

**Critical appraisal checklist scoring guidance:**

*Note: For each item, one point was deducted from total score of three for every factor potentially contributing to bias present.*

1. **Was the sample frame appropriate to address the target population?**

***Ideal methods:***

- *A study enrolling all eligible hospital admissions through census data*
- *General ward admissions*
- *For studies enrolling specific disease sub-groups (e.g. anemia, pneumonia, or diarrhea, etc.), the hospitals should capture participants from the general admissions population*

***Factors potentially contributing to bias:***

- *A study which enrols participants from specialty wards (like HIV, Tuberculosis, or nutritional wards).*
- *For a study requiring consent, if there are a high proportion of refusals, the sampling frame may be biased*

1. **Were study participants recruited in an appropriate way?**

***Ideal methods:***

- *The study uses all available census data on the target population*
- *A study which enrols children prospectively from a hospital using random probabilistic sampling*

***Factors potentially contributing to bias:***

- *The study prospectively enrols children during pre-specified study hours (e.g. 8am-5pm only)*
- *An inappropriate alternative sampling method is used (e.g. convenience sampling)*

1. **Were the study subjects and setting described in detail?**

***Ideal methods:***

- *The study provides a sufficient description of both the study subjects (e.g. age distribution, male/female sex proportion, or prevalence of comorbidities) and the study setting (including, but not limited to, hospital size(s), location(s), annual admissions)*

***Factors potentially contributing to bias:***

- *The study does not provide a detailed description for either the study subjects or the study setting*

1. **Were valid methods used for the identification of the condition and risk factors?**

***Ideal methods:***

- *For the condition: the condition defining the overall cohort is specifically defined (e.g. for a cohort of pneumonia admissions, the criteria for pneumonia disease classification is explicitly stated)*
- *For post-discharge mortality risk factors: the risk factors are explicitly described (e.g. if malnutrition is identified as a risk factor, the diagnostic criteria for its classification is explicitly stated).*

***Factors potentially contributing to bias:***

- *No specific criteria was followed when enrolling the population condition*
- *Risk factors are listed without proper description of their classification*
- *Non-validated instruments, or inappropriate* measurement techniques were used in the collection of risk factor measurements, without justification

1. **Were the condition and risk factors measured in a standard, reliable way for all participants?**

***Ideal methods:***

- *The condition or risk factors are measured prospectively*
- *Conditions or risk factors are measured using appropriate instruments (thermometers vs. febrile symptoms).*

***Factors potentially contributing to bias:***

- *Risk factors are measured using unknown/variety data collection instruments within or across study sites (often the case with retrospective data or routine clinical data*)

1. **Was there appropriate statistical analysis?**

***Ideal methods:***

- *The number enrolled for discharge follow-up (denominator), and the number who died post-discharge (numerator) are clearly stated*
- *The methods section provides sufficient detail to determine how the outcome variables were measured*

***Factors potentially contributing to bias:***

- *There is uncertainty in either the numerator or denominator (unsure if denominator are those discharged alive vs admitted).*
- *Uncertainty in how losses to follow-up were accounted for*
- *Uncertainty in how missing data was dealt with in risk factor analysis*

1. **Was the response rate adequate, and if not, was the low response rate managed appropriately?**

***Scoring guidance:***

*Low Risk: Response rate >90%*

*Moderate Risk: Response rate between 80-90%*

*High Risk: Response rate between <80% or not mentioned*

## Table S8. Summary of risk of bias assessment results.

| **Reference** | **1. Was the sample frame appropriate to address the target population?** | **2. Were study participants sampled in an appropriate way?** | **3. Were the study subjects and the setting described in detail?** | **4. Were valid methods used for the identification of the condition and risk factors?** | **5. Were the condition and risk factors measured in a standard, reliable way for all participants?** | **6. Was there appropriate statistical analysis?** | **7. Was the response rate adequate?*** | **Total Score** |
| --- | --- | --- | --- | --- | --- | --- | --- | --- |
| Ashraf et al., 2012 | 3 | 3 | 3 | 3 | 3 | 3 | 3 | 21 |
| Berkley et al., 2016 | 3 | 3 | 3 | 3 | 3 | 3 | 3 | 21 |
| Biai et al., 2007 | 3 | 3 | 3 | 3 | 3 | 3 | 3 | 21 |
| Brim et al., 2017 | 3 | 3 | 3 | 3 | 3 | 3 | 2 | 20 |
| Bwakura‐Dangarembizi et al., 2021a | 3 | 3 | 3 | 3 | 3 | 3 | 3 | 21 |
| CHAIN Network, 2022 | 3 | 3 | 3 | 3 | 3 | 3 | 3 | 21 |
| Chapagain et al., 2022 | 3 | 3 | 3 | 3 | 2 | 3 | 3 | 20 |
| Chhibber et al., 2015 | 3 | 3 | 3 | 3 | 3 | 3 | 1 | 19 |
| Chisti et al., 2014 | 2 | 3 | 3 | 3 | 3 | 3 | 2 | 19 |
| Grenov et al., 2017 | 2 | 3 | 3 | 3 | 3 | 3 | 3 | 20 |
| Hamaluba et al., 2021 | 3 | 3 | 3 | 3 | 3 | 3 | 3 | 21 |
| Hau et al., 2018 | 3 | 3 | 3 | 3 | 3 | 3 | 1 | 19 |
| Hawkes et al., 2015 | 3 | 3 | 3 | 3 | 3 | 3 | 2 | 20 |
| Hennart et al., 1987 | 3 | 1 | 2 | 3 | 3 | 3 | 1 | 16 |
| Islam et al., 1996 | 2 | 2 | 3 | 2 | 3 | 3 | 1 | 16 |
| Kerac et al., 2014 | 2 | 3 | 3 | 3 | 3 | 3 | 2 | 19 |
| Kwambai et al., 2020 | 3 | 3 | 3 | 3 | 3 | 3 | 3 | 21 |
| Madrid et al., 2019 | 3 | 3 | 3 | 3 | 2 | 3 | 3 | 20 |
| Maitland et al., 2019 | 3 | 3 | 3 | 3 | 3 | 3 | 3 | 21 |
| Masoza et al., 2022 | 3 | 3 | 3 | 3 | 3 | 3 | 3 | 21 |
| Moisi et al. (2011) | 3 | 3 | 3 | 2 | 2 | 3 | 1 | 17 |
| Mukasa et al., 2021 | 3 | 3 | 3 | 2 | 2 | 3 | 3 | 19 |
| Mwangome et al., 2017 | 3 | 3 | 3 | 3 | 2 | 3 | 3 | 20 |
| Mwene-Batu et al., 2020 | 3 | 3 | 3 | 3 | 2 | 3 | 1 | 18 |
| Namazzi et al., 2022 | 3 | 3 | 3 | 3 | 3 | 3 | 3 | 21 |
| Ngari et al., 2017 | 3 | 3 | 3 | 3 | 2 | 3 | 3 | 20 |
| Ngari et al., 2020 | 3 | 3 | 3 | 3 | 2 | 3 | 3 | 20 |
| Nkosi-Gondwe et al., 2021b | 3 | 3 | 3 | 3 | 3 | 3 | 3 | 21 |
| Olupot-Olupot et al., 2014 | 3 | 3 | 3 | 3 | 3 | 3 | 3 | 21 |
| Opoka et al., 2019 | 3 | 3 | 3 | 3 | 2 | 3 | 3 | 20 |
| Ouma et al., 2020 | 3 | 3 | 3 | 3 | 3 | 3 | 3 | 21 |
| Page et al., 2017 | 3 | 3 | 3 | 3 | 3 | 3 | 2 | 20 |
| Pavlinac et al., 2021 | 3 | 2 | 3 | 3 | 3 | 3 | 1 | 18 |
| Phiri et al., 2008 | 3 | 3 | 3 | 3 | 3 | 3 | 2 | 20 |
| Phiri et al., 2012 | 3 | 3 | 3 | 3 | 3 | 3 | 3 | 21 |
| Roy et al., 1983 | 2 | 1 | 3 | 3 | 3 | 3 | 1 | 16 |
| Shahrin et al., 2020 | 2 | 3 | 3 | 3 | 3 | 3 | 3 | 20 |
| Stanton et al., 1986 | 2 | 3 | 3 | 2 | 2 | 3 | 3 | 18 |
| Talbert et al., 2019 | 3 | 3 | 3 | 3 | 2 | 3 | 3 | 20 |
| Tomczyk et al., 2019 | 3 | 3 | 3 | 3 | 2 | 3 | 1 | 18 |
| Veirum et al., 2007 | 3 | 3 | 3 | 3 | 3 | 3 | 1 | 19 |
| Villamor et al., 2005 | 3 | 3 | 3 | 3 | 3 | 3 | 2 | 20 |
| West et al., 1999 | 3 | 2 | 3 | 2 | 3 | 3 | 1 | 17 |
| Wiens et al., 2015 | 3 | 3 | 3 | 3 | 3 | 3 | 3 | 21 |
| Wiens et al., 2023 | 3 | 3 | 3 | 3 | 3 | 3 | 3 | 21 |
| Zucker et al., 1996 | 3 | 3 | 3 | 3 | 3 | 3 | 3 | 21 |

Scoring: 1=High risk of bias; 2=Moderate risk of bias; 3=Low risk of bias

*Low risk: Response rate >90%; Moderate risk: Response rate 80-90%; High risk: Response rate <80%.

# Publication Bias Assessment

## Figure S1. Six-month post-discharge mortality funnel plot.


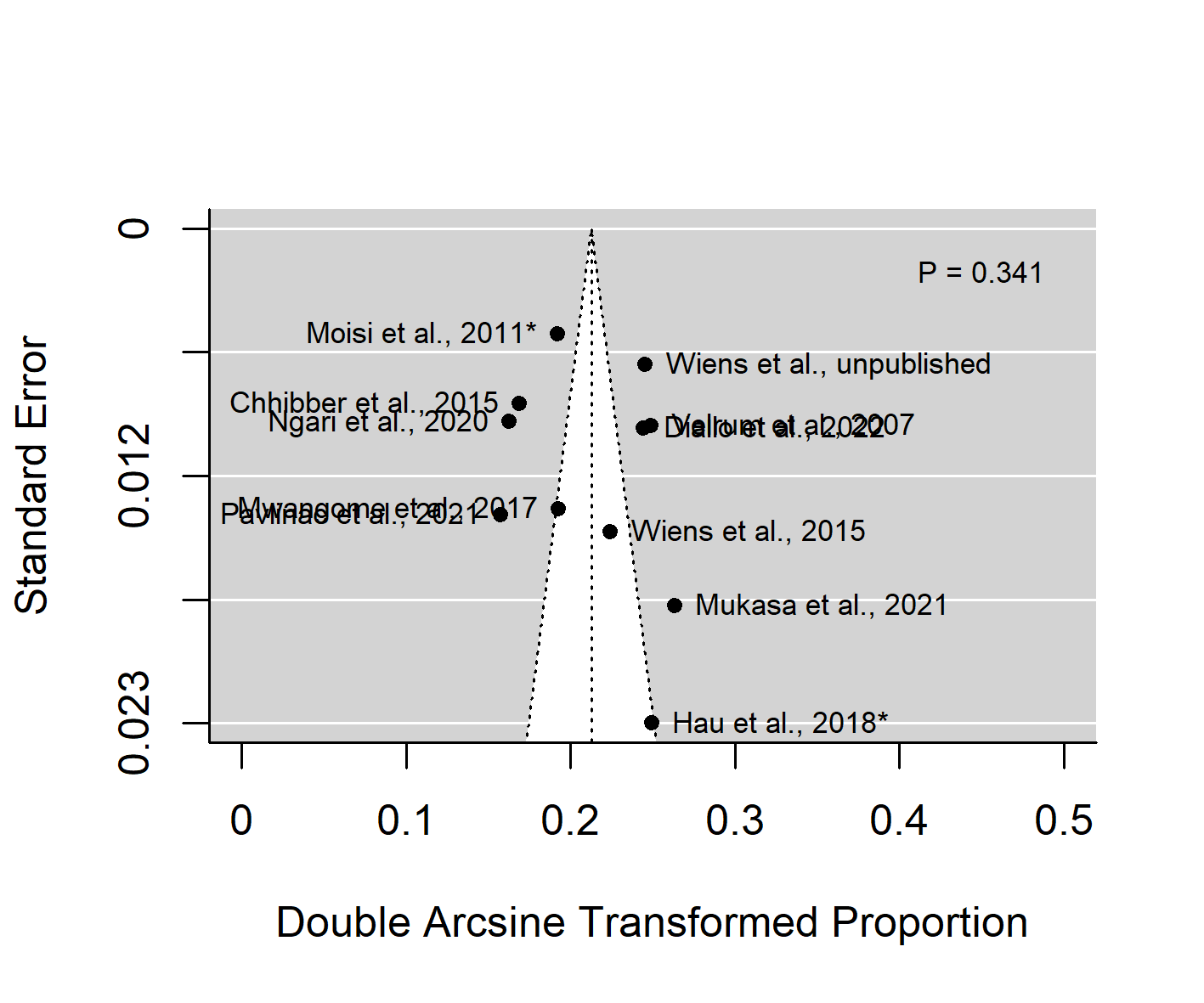


## Figure S2. In-hospital mortality funnel plot


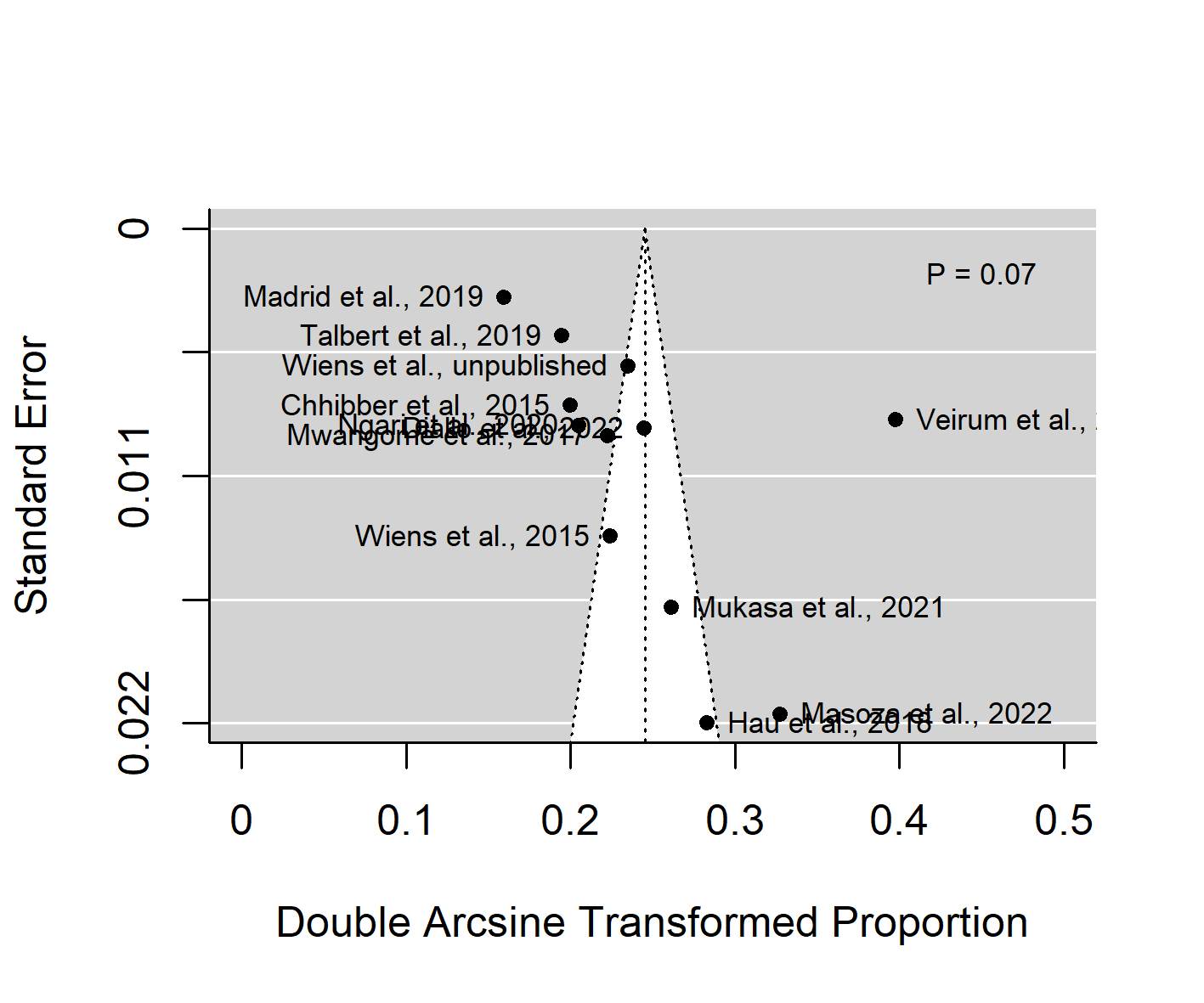


# Supplementary Results

## **Six Month Post-Discharge Mortality Estimates**

### Figure S3. Proportion of six-month post-discharge mortality among studies of general acute illness.


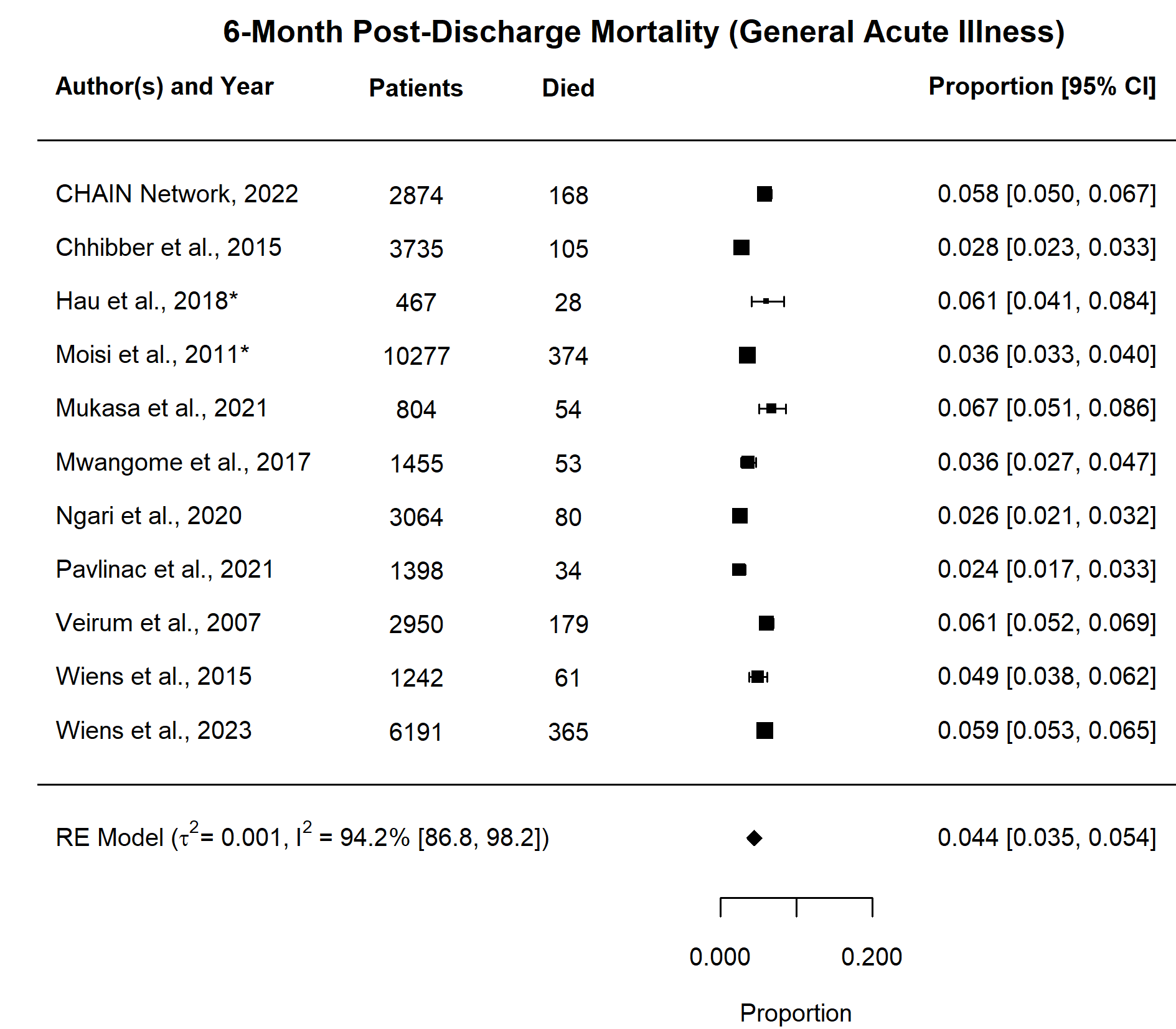


### Figure S4. Proportion of six-month post-discharge mortality among population sub-groups.


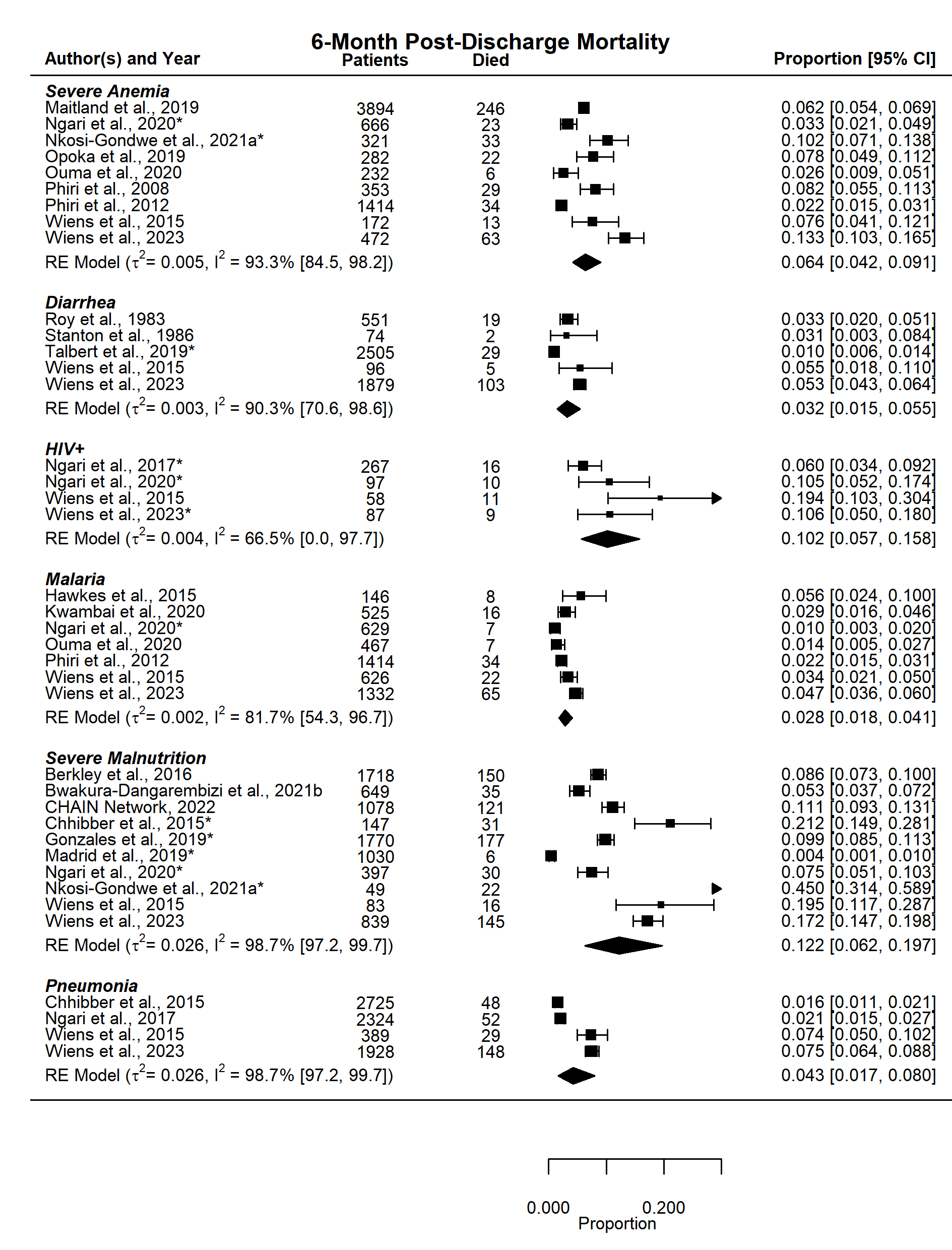


*Estimate extracted from survival curve data

## **Twelve Month Post-Discharge Mortality Estimates**

### Table S9. 12-month post-discharge mortality fixed and random effects estimates.

| **Disease Subgroup** | **Studies, N** | **Patients, N** | **Fixed Effects, Proportion (95% CI)** | **Random Effects, Proportion (95% CI)** |
| --- | --- | --- | --- | --- |
| General acute illness | 6 | 28552 | 3.9 (3.68, 4.13) | 5.08 (3.05, 7.59) |
| Severe anemia | 6 | 1743 | 8.09 (6.78, 9.5) | 8.45 (4.02, 14.13) |
| Diarrhea | 5 | 3642 | 2.51 (2.01, 3.06) | 3.64 (2.23, 5.34) |
| HIV+ | 2 | 364 | 18.3 (14.46, 22.47) | 17.99 (13.37, 23.11) |
| Malaria | 5 | 1871 | 1.31 (0.74, 2) | 1.36 (0.31, 2.88) |
| Moderate malnutrition | 2 | 1001 | 2.46 (1.52, 3.57) | 5.04 (0.11, 15.22) |
| Severe malnutrition | 7 | 4075 | 12.39 (11.38, 13.44) | 11.93 (7.45, 17.26) |
| Pneumonia | 4 | 6561 | 4.04 (3.57, 4.53) | 4.91 (2.91, 7.39) |

### Figure S5. 12-Month post-discharge mortality among general acute illness.


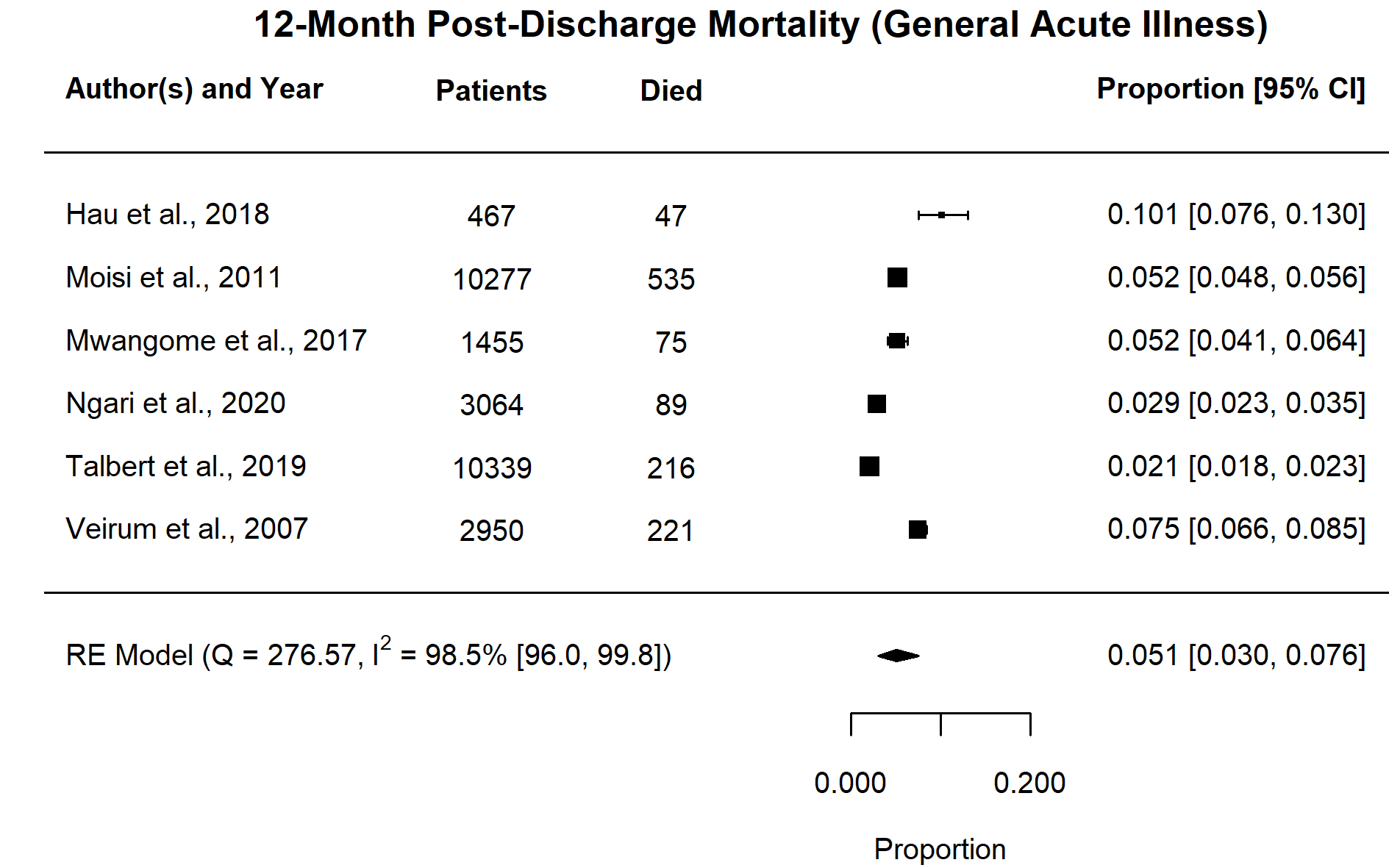


### Figure S6. 12-Month post-discharge mortality among population sub-groups.


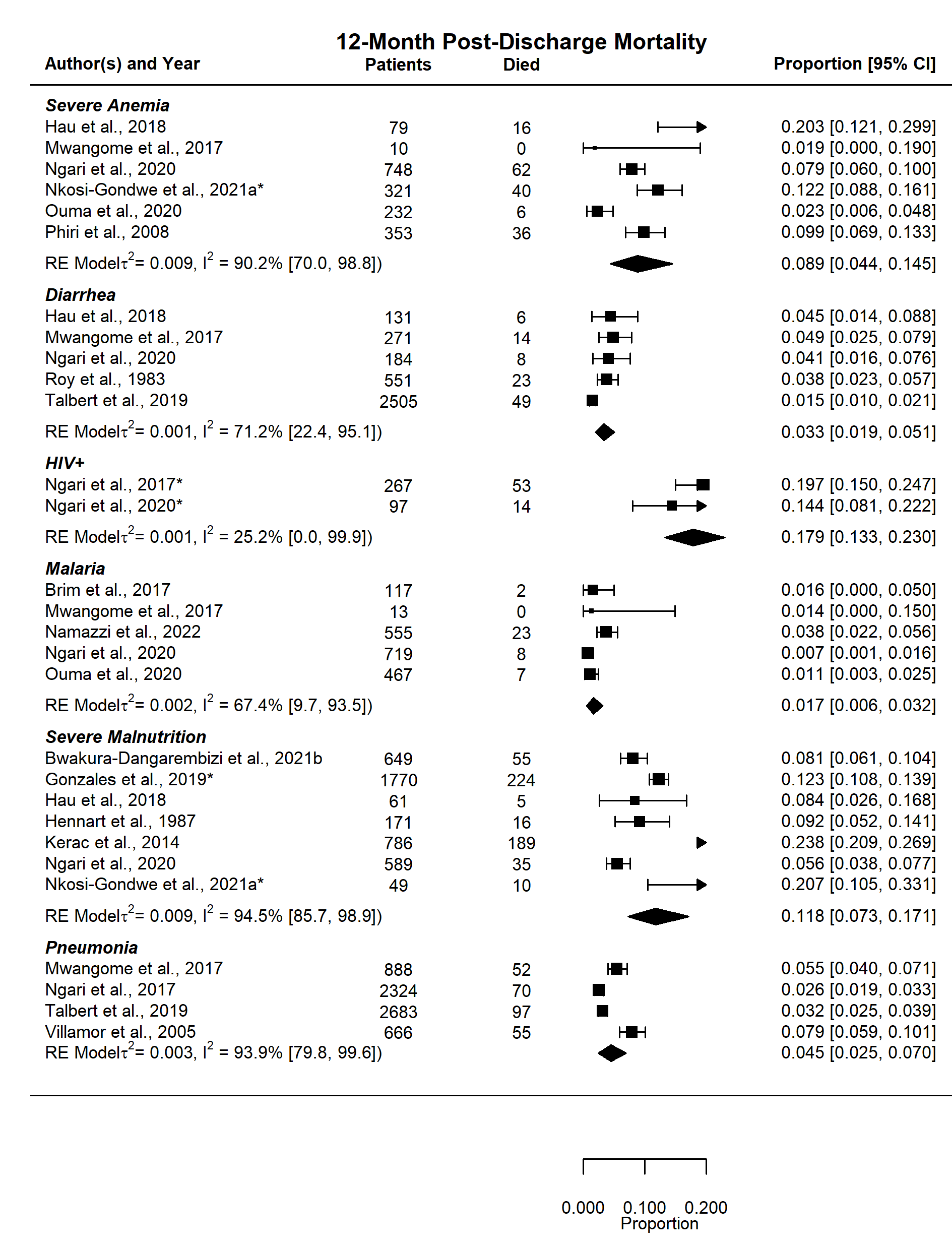


*Estimate extracted from survival curve data

## **Hazard Ratio Pooling**

### Table S10. Fixed and random effects pooled hazard ratios.

| **Variable** | **Studies, N** | **Fixed Effects,**  **HR (95% CI)** | **Random Effects,**  **HR (95% CI)** |
| --- | --- | --- | --- |
| Age | 4 | 0.99 (0.98, 1) | 0.99 (0.98, 1) |
| Anemia | 5 | 3.28 (2.85, 3.78) | 1.93 (1.02, 3.65) |
| Bacteremia | 5 | 2.92 (2.62, 3.26) | 2.17 (1.4, 3.37) |
| Diarrhea | 6 | 1.33 (1.17, 1.5) | 1.14 (0.72, 1.81) |
| Edema | 6 | 1.42 (1.19, 1.7) | 1.5 (0.92, 2.45) |
| HIV | 8 | 3.52 (3.26, 3.81) | 3.06 (1.75, 5.32) |
| Hypoxia | 7 | 2 (1.73, 2.3) | 2 (1.73, 2.3) |
| Impaired Consciousness | 5 | 2.75 (2.71, 2.79) | 2.05 (1.53, 2.75) |
| Increased RR | 4 | 1.88 (1.79, 1.97) | 1.6 (1.29, 1.99) |
| Malaria | 8 | 0.56 (0.49, 0.64) | 0.59 (0.41, 0.85) |
| Moderate Malnutrition | 8 | 2.06 (1.92, 2.22) | 2.06 (1.91, 2.23) |
| Severe Malnutrition | 10 | 3.96 (3.51, 4.47) | 3.68 (2.91, 4.67) |
| Some Maternal Schooling | 4 | 0.7 (0.6, 0.81) | 0.85 (0.47, 1.55) |
| MUAC, per decrease in mm | 6 | 1.04 (1.03, 1.04) | 1.04 (1.03, 1.05) |
| Prior Admission | 4 | 2.59 (2.35, 2.86) | 2.13 (1.07, 4.24) |
| Sex (Male) | 11 | 1.96 (1.94, 1.98) | 1.03 (0.83, 1.28) |
| Unplanned Discharge | 5 | 4.32 (3.74, 4.99) | 4.24 (2.67, 6.74) |

### Figure S7. Pooled hazard ratios (random-effects) for post-discharge mortality, adjusted estimates only.


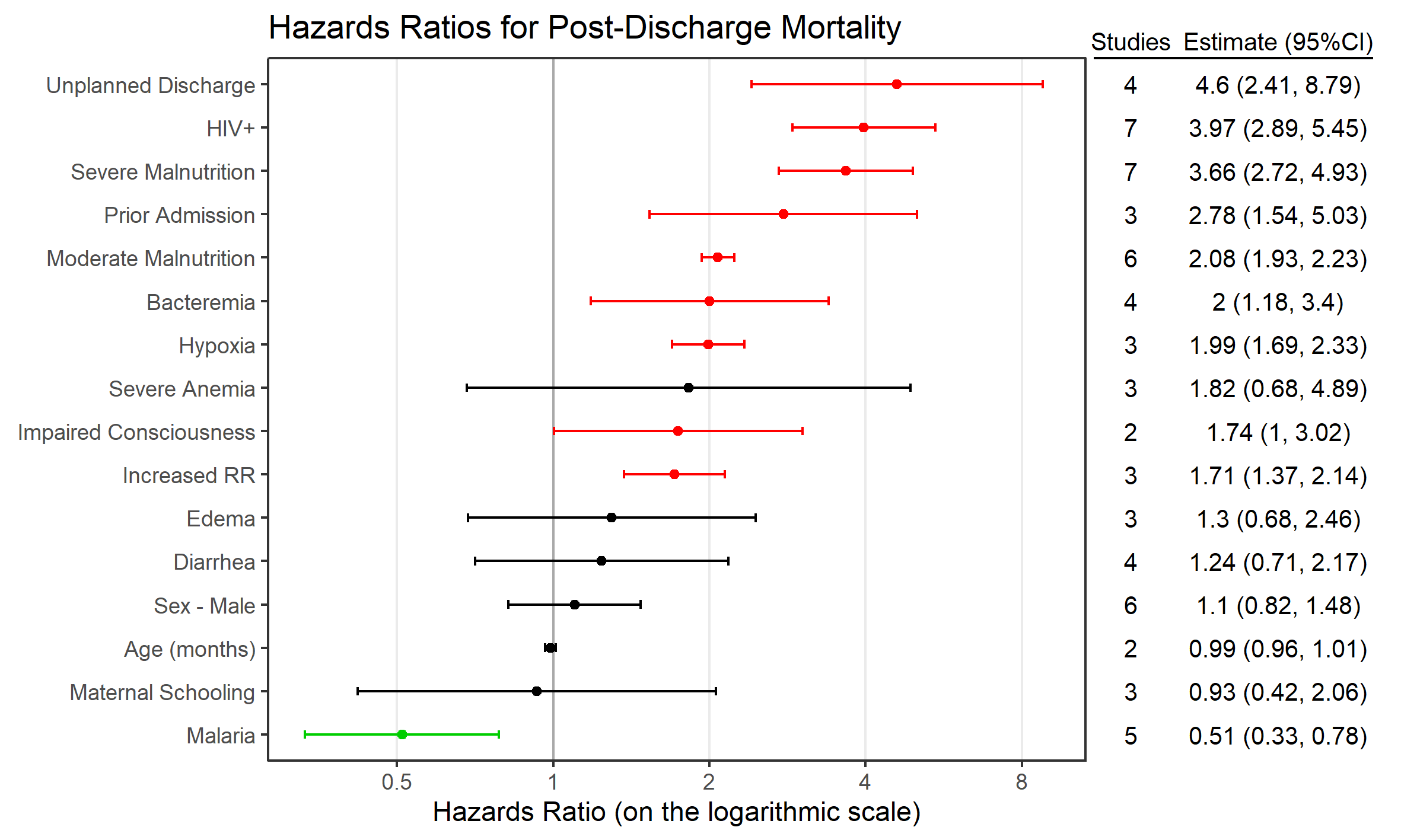


### Figure S8. Pooled hazard ratios (random-effects) for post-discharge mortality, unadjusted estimates only.

**
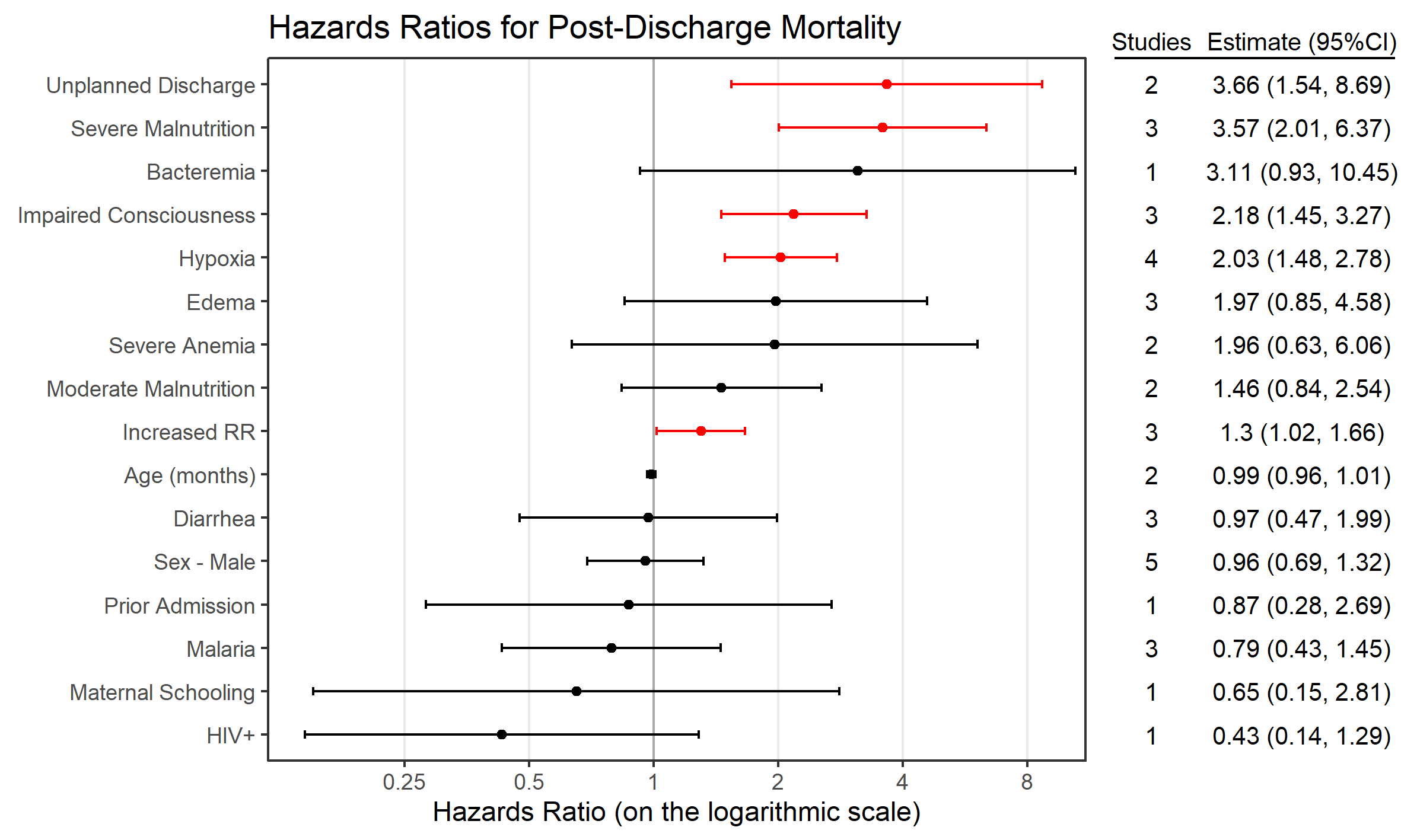
**

## **Survival Curve Analysis**

### Table S11. Median time to death post-discharge among disease sub-groups.

| **Disease Sub-group** | **Time to death (in weeks), Median (95% CI)** |
| --- | --- |
| General Acute Illness | 7.4 (6.4, 8.8) |
| Severe Anemia | 8.5 (4.9, 13.5) |
| Diarrhea | 3.3 |
| HIV+ | 9.2 (4, 13.3) |
| Malaria | 3.7 |
| Moderate Malnutrition | 6.3 (3.8, 9.1) |
| Severe Malnutrition | 6.7 (4.1, 9.8) |
| Pneumonia | 7.4 |

NA=Not available, insufficient data

### Table S12. Median time to death post-discharge among age sub-groups.

| **Age sub-group** | **Time to death (in weeks), Median (95% CI)** |
| --- | --- |
| <6 Months | 7.8 (3.4, 15.8) |
| 6-23 Months | 7 (3.1, 13.4) |
| 24-59 Months | 9.4 (3.6, 15.7) |
| >60 Months | 6 (2.5, 10.2) |

NA=Not available, insufficient data

### Figure S9. Individual study mortality curves (1 minus survival) across all sub-groups.


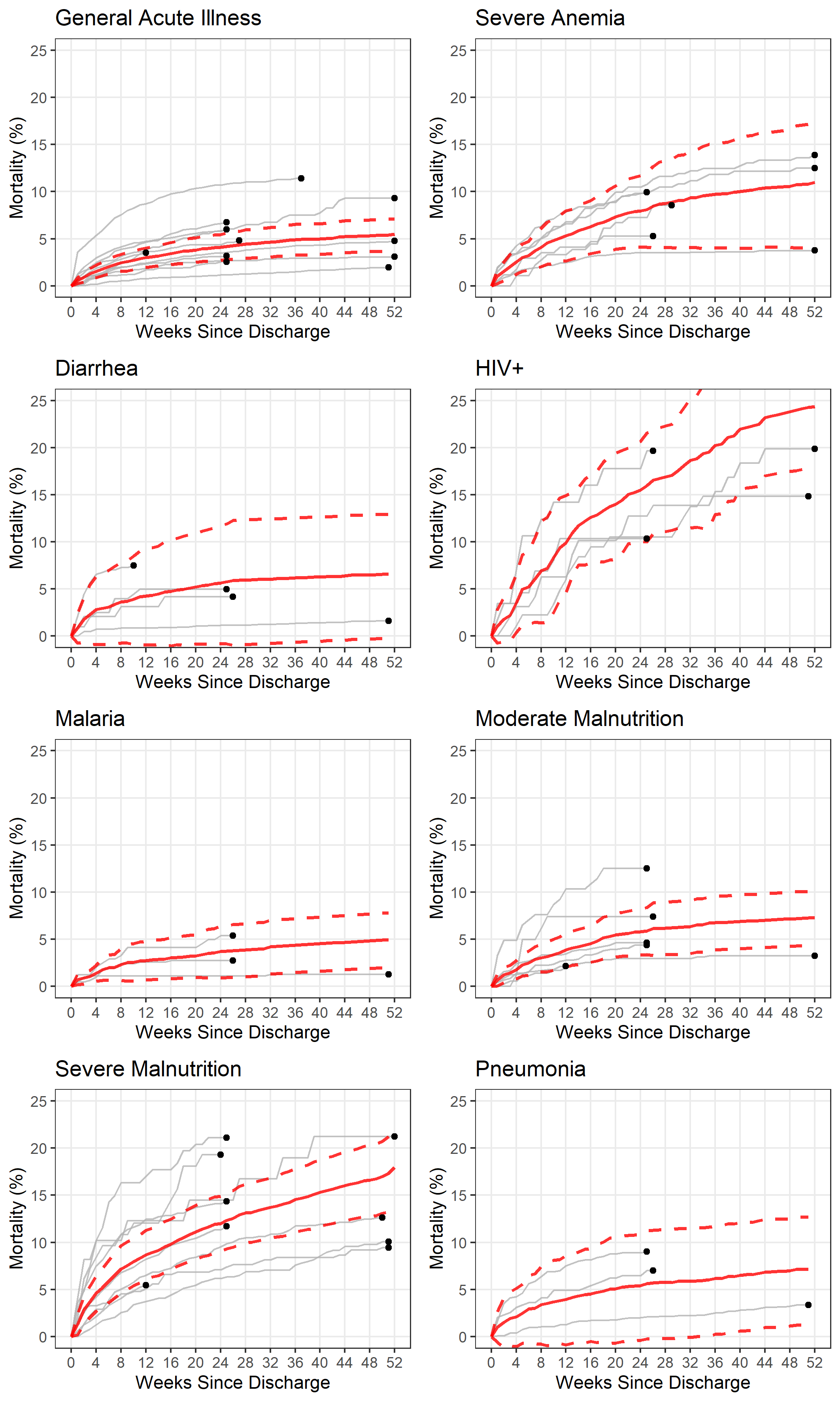


Grey line: survival curves from individual studies with the black point indicating their endpoint

Red solid line: pooled mortality curve

Red dotted line: 95% confidence interval for pooled mortality curve

### Figure S10. Individual study mortality curves (1 minus survival) across age groups.

**
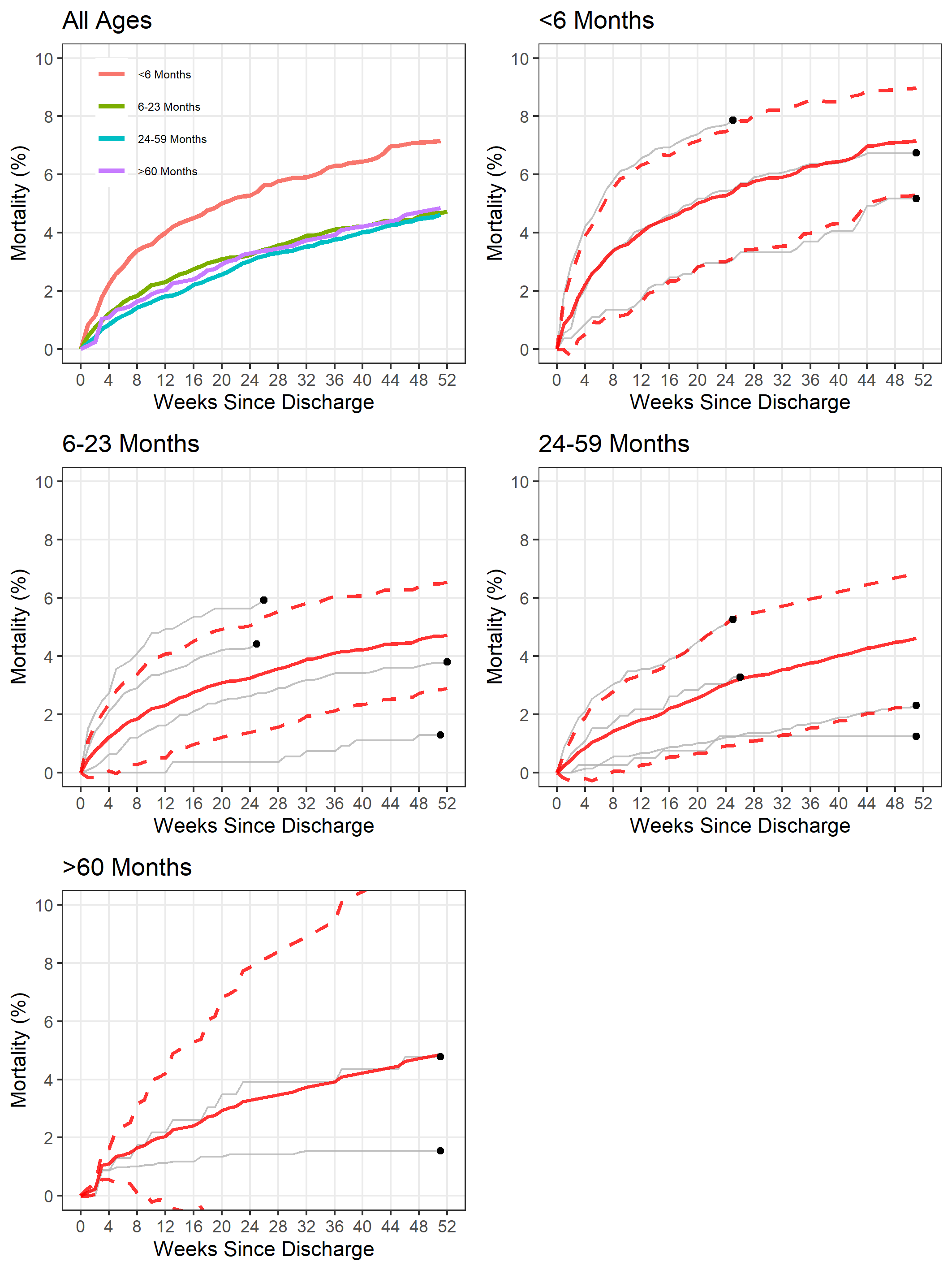
**

Grey line: survival curves from individual studies with the black point indicating their endpoint

Red solid line: pooled mortality curve

Red dotted line: 95% confidence interval for pooled mortality curve

## **In-Hospital Mortality**

### Table S13. In-hospital mortality fixed and random effects estimates.

| **Disease Subgroup** | **Studies, N** | **Patients, N** | **Died, N** | **Fixed Effects, Proportion (95% CI)** | **Random Effects, Proportion (95% CI)** |
| --- | --- | --- | --- | --- | --- |
| General Acute Illness | 12 | 63307 | 2746 | 4.03 (3.88, 4.18) | 5.86 (4.24, 7.73) |
| Severe Anemia | 8 | 6427 | 240 | 3.24 (2.81, 3.7) | 5.22 (2.63, 8.6) |
| Diarrhea | 6 | 5247 | 259 | 4.75 (4.18, 5.36) | 4.75 (4.18, 5.36) |
| HIV+ | 1 | 49 | 14 | 28.57 (16.67, 42.13) | 28.57 (16.67, 42.13) |
| Malaria | 6 | 4373 | 251 | 5.62 (4.95, 6.32) | 5.98 (4.64, 7.48) |
| Moderate Malnutrition | 4 | 2723 | 137 | 4.73 (3.94, 5.57) | 5.07 (2.89, 7.79) |
| Severe Malnutrition | 10 | 7399 | 731 | 9.05 (8.4, 9.72) | 9.56 (6.51, 13.11) |
| Pneumonia | 5 | 7628 | 315 | 3.92 (3.49, 4.37) | 3.19 (1.16, 6.12) |
| Other | 2 | 615 | 92 | 14.4 (11.72, 17.31) | 11.4 (2.32, 25.87) |

### Figure S11. In-hospital mortality random effects estimates among general acute illness.


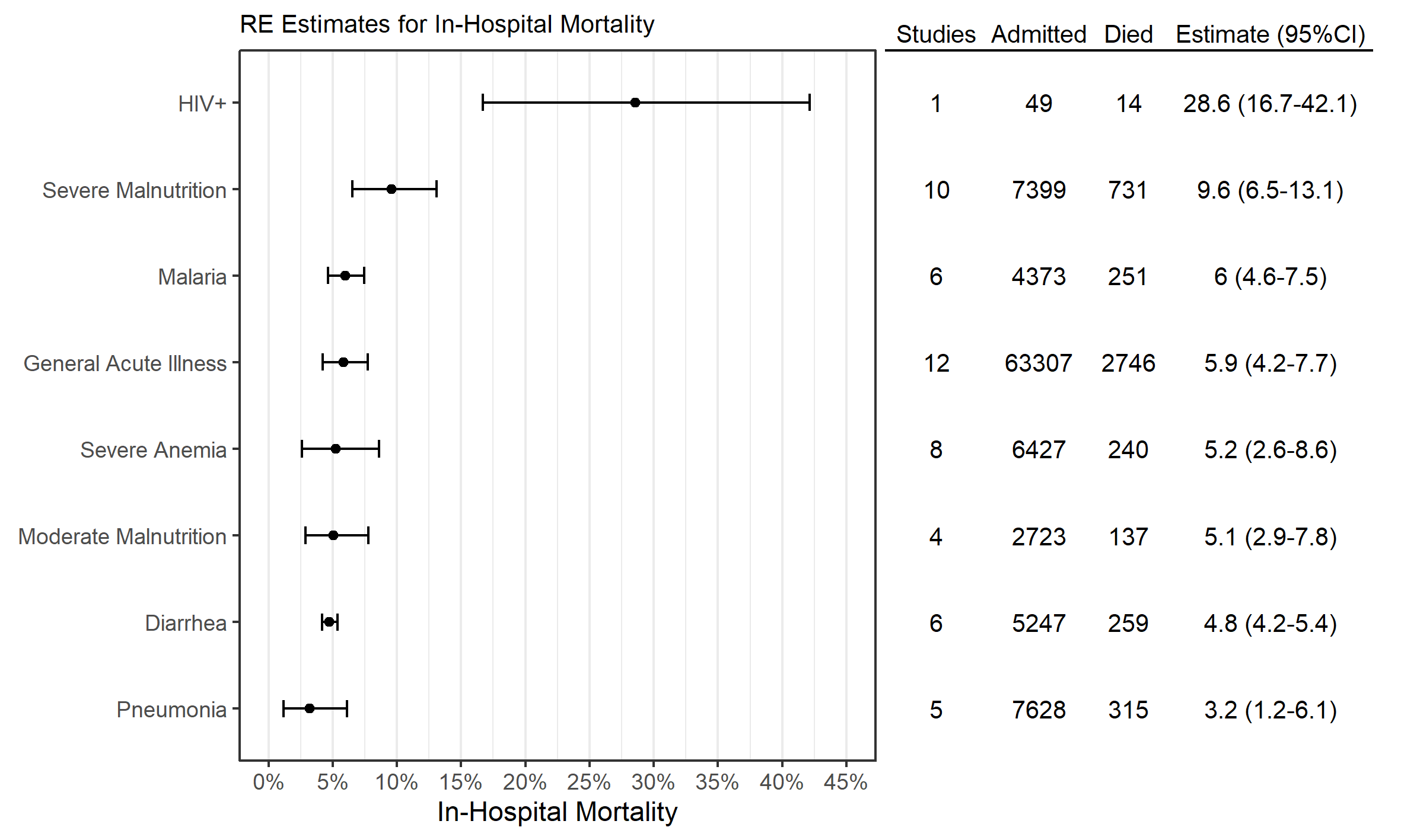


### Figure S12. In-hospital mortality among general acute illness.


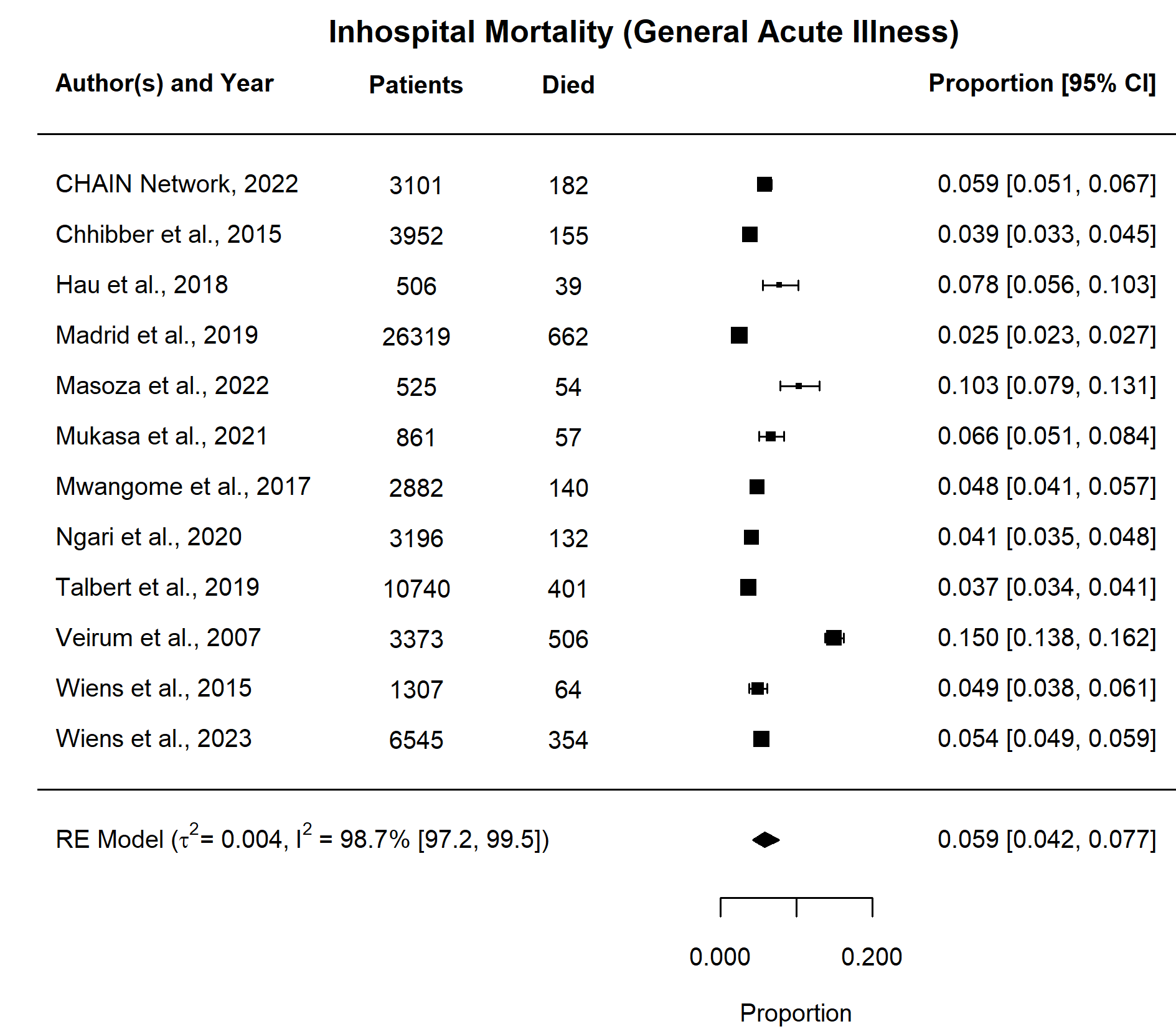


### Figure S13. In-hospital mortality among disease sub-groups.


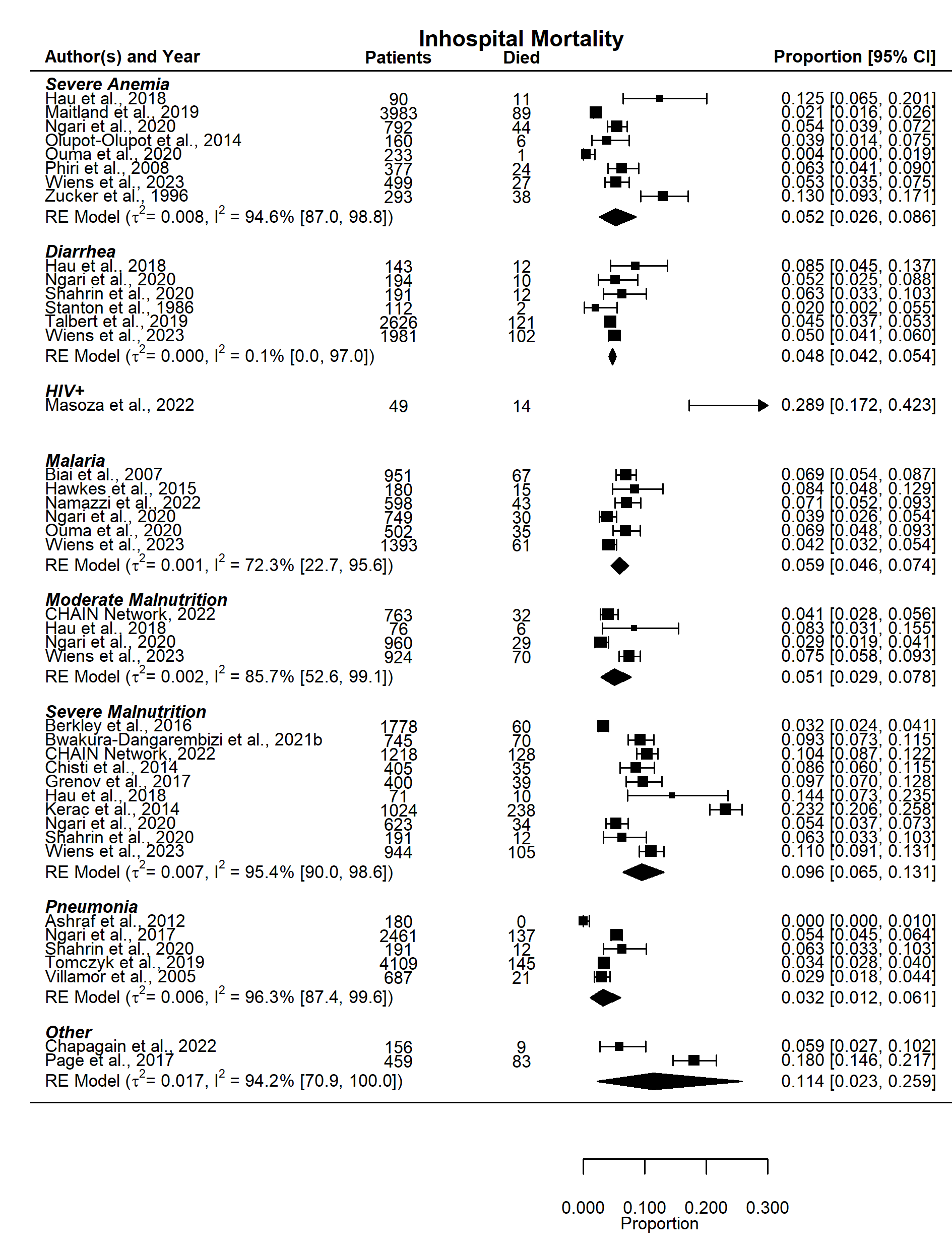


### Figure S14. Difference between post-discharge in-hospital mortality rates.


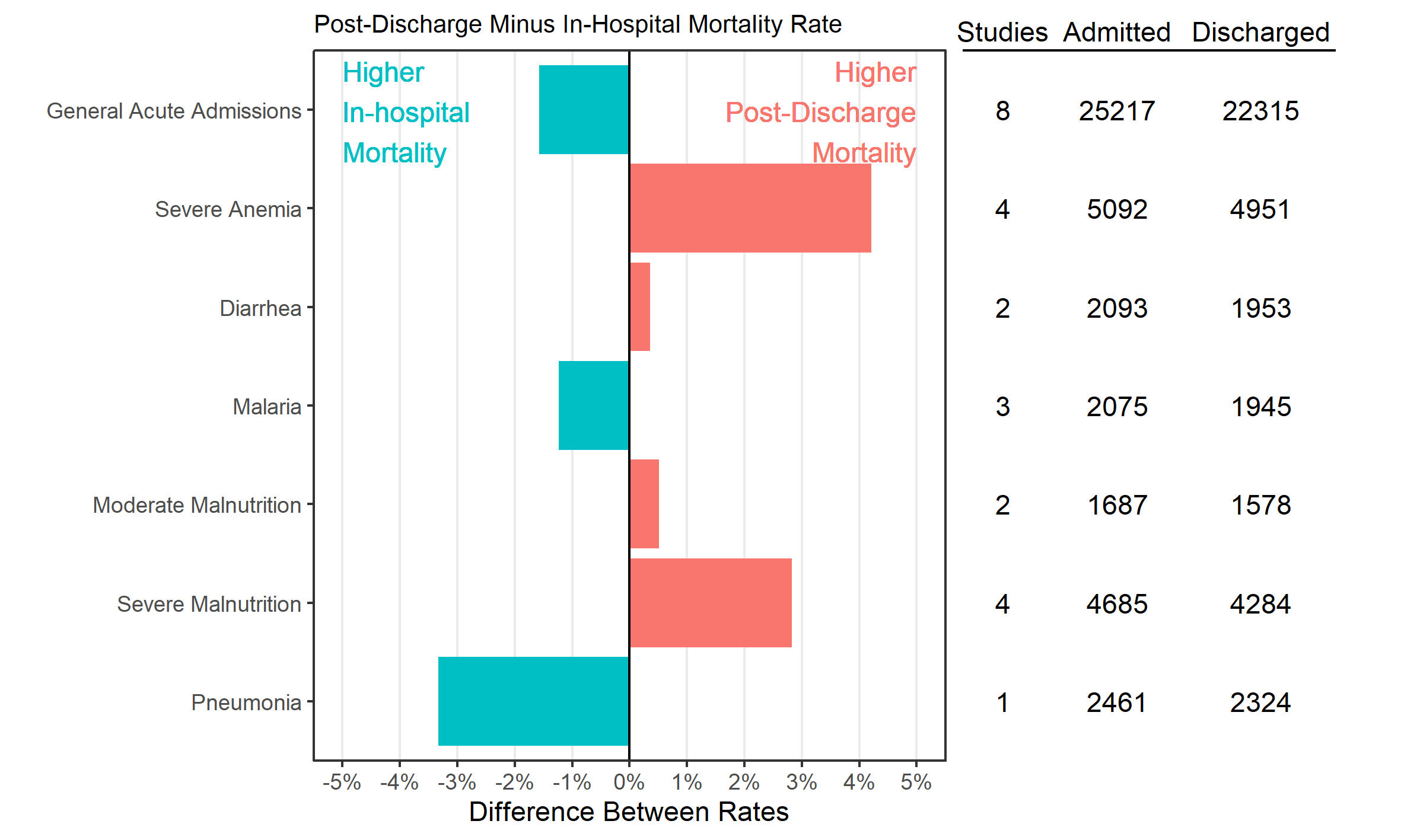


### Figure S15. In-hospital versus post-discharge proportion of all deaths.


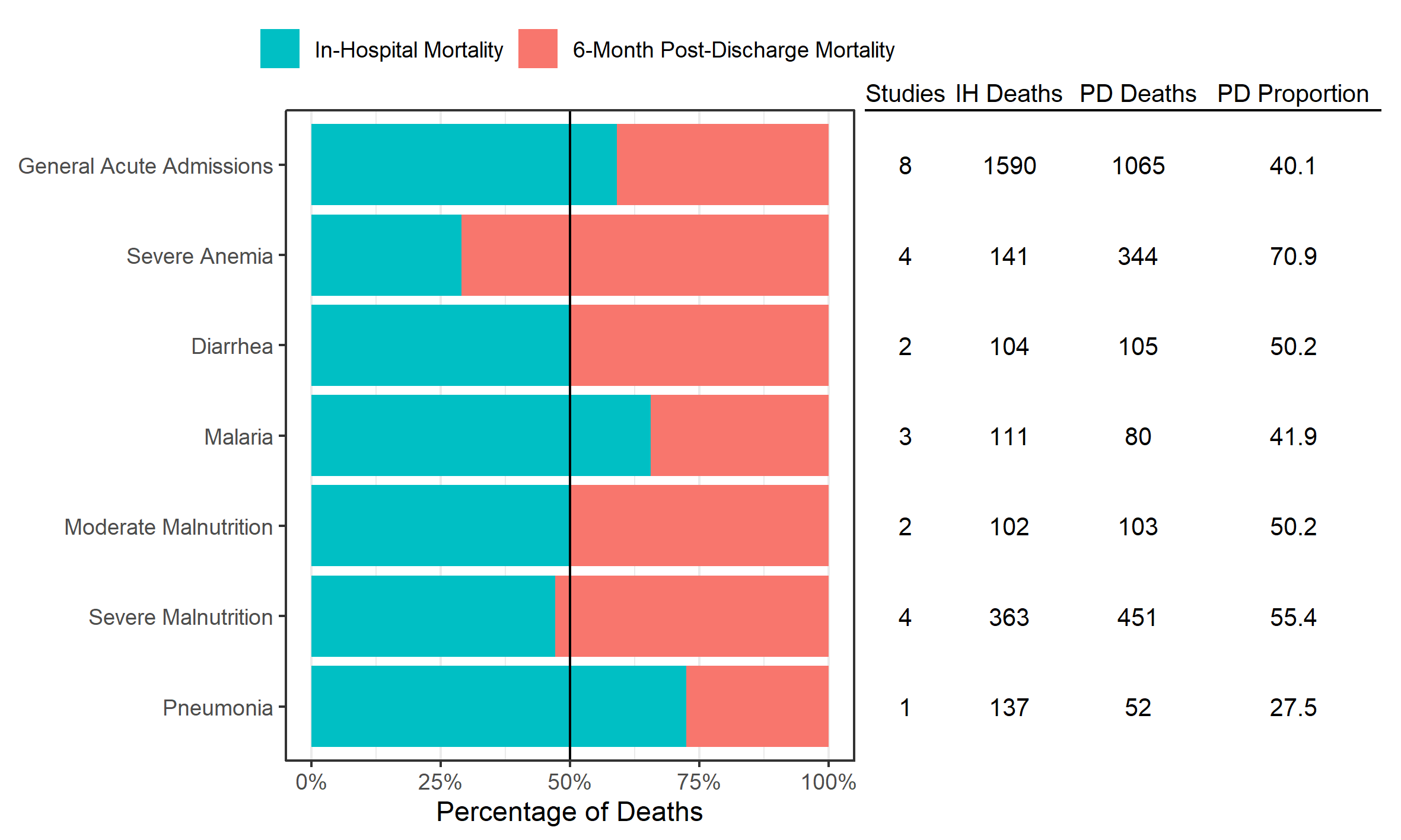


### Figure S16. Post-discharge mortality versus in-hospital mortality proportion scatterplot for general acute illness studies.


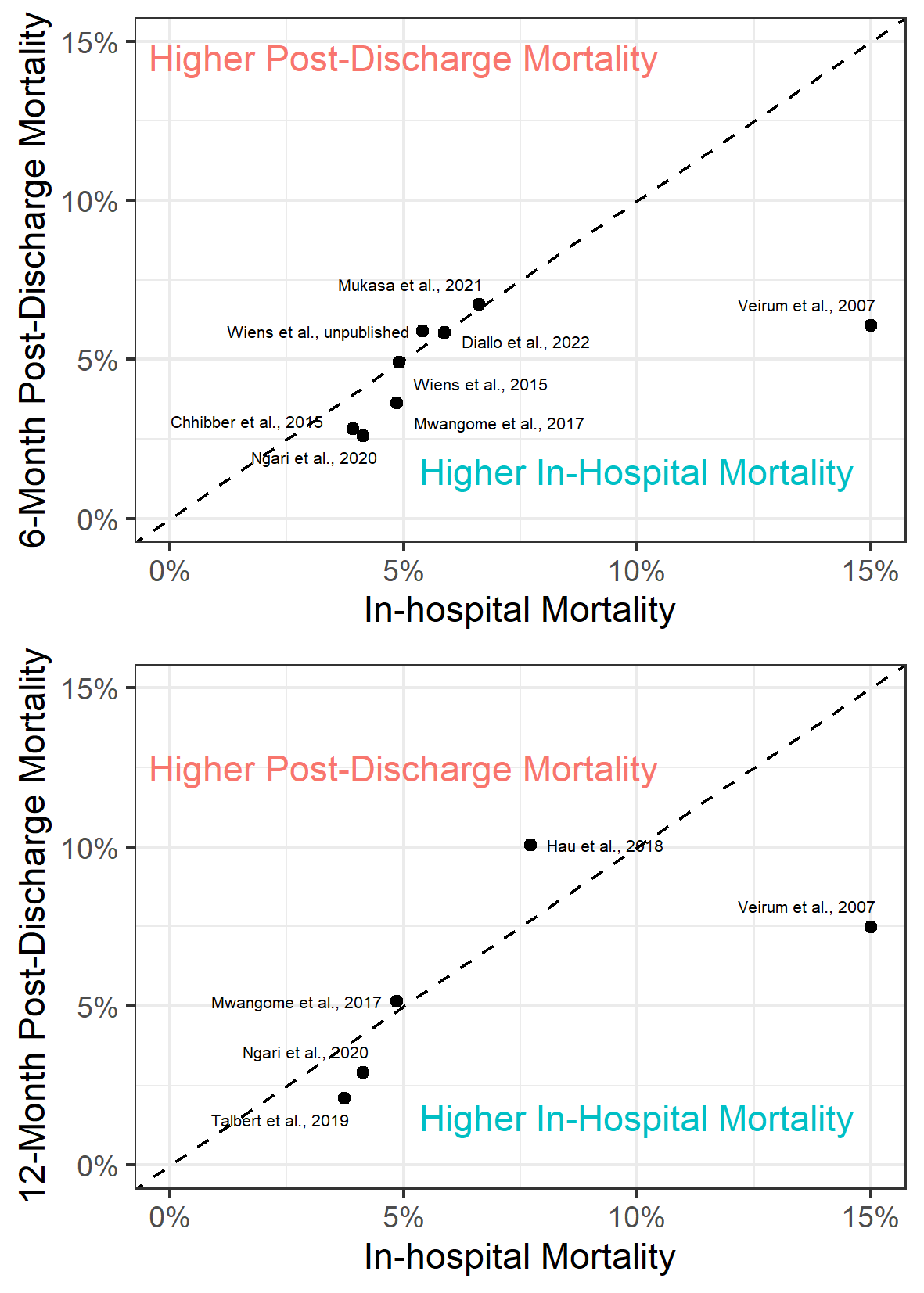


## **Sensitivity Analyses**

### Table S14. 6-month post-discharge mortality fixed and random effects estimates for studies whose recruitment end date was after January 1st 2010.

| **Disease Subgroup** | **Studies, N** | **Patients, N** | **Fixed Effects Estimate, Proportion (95% CI)** | **Random Effects Estimate, Proportion (95% CI)** |
| --- | --- | --- | --- | --- |
| General acute illness | 8 | 20426 | 4.23 (3.96, 4.51) | 4.1 (3.08, 5.27) |
| Severe anemia | 6 | 5807 | 6.65 (6.01, 7.31) | 7.76 (5.13, 10.86) |
| Diarrhea | 3 | 4480 | 2.53 (2.08, 3.03) | 3.39 (0.82, 7.47) |
| HIV+ | 4 | 509 | 8.56 (6.21, 11.21) | 10.19 (5.67, 15.78) |
| Malaria | 4 | 3112 | 3.36 (2.75, 4.02) | 3.01 (1.57, 4.88) |
| Moderate malnutrition | 5 | 2560 | 4.56 (3.76, 5.42) | 5.73 (2.13, 10.83) |
| Severe malnutrition | 8 | 5341 | 8.3 (7.56, 9.07) | 13.65 (6.08, 23.56) |
| Pneumonia | 4 | 7366 | 3.35 (2.95, 3.78) | 4.29 (1.67, 8.02) |

### Table S15. 6-month post-discharge mortality fixed and random effects estimates with RCTs excluded.

| **Disease Subgroup** | **Studies, N** | **Patients, N** | **Fixed Effects Estimate, Proportion (95% CI)** | **Random Effects Estimate, Proportion (95% CI)** |
| --- | --- | --- | --- | --- |
| General acute illness | 10 | 33059 | 4.32 (4.11, 4.55) | 4.64 (3.71, 5.66) |
| Severe anemia | 6 | 2177 | 6.66 (5.64, 7.76) | 6.76 (3.94, 10.26) |
| Diarrhea | 5 | 5105 | 2.55 (2.12, 3.03) | 3.21 (1.49, 5.50) |
| HIV+ | 4 | 509 | 8.56 (6.21, 11.21) | 10.19 (5.67, 15.78) |
| Malaria | 4 | 3054 | 3.08 (2.49, 3.73) | 2.60 (1.15, 4.59) |
| Moderate malnutrition | 5 | 2560 | 4.56 (3.76, 5.42) | 5.73 (2.13, 10.83) |
| Severe malnutrition | 8 | 5993 | 4.56 (3.76, 5.42) | 5.73 (2.13, 10.83) |
| Pneumonia | 4 | 7366 | 8.19 (7.50, 8.9) | 10.05 (5.20, 16.24) |
